# Breakfast Habits of a Representative Sample of the Spanish Child and Adolescent Population (The ENALIA Study): Association with Diet Quality

**DOI:** 10.3390/nu12123772

**Published:** 2020-12-08

**Authors:** Esther Cuadrado-Soto, Ana M. López-Sobaler, Ana Isabel Jiménez-Ortega, Laura M. Bermejo, Aránzazu Aparicio, Rosa M. Ortega

**Affiliations:** 1 Department of Nutrition and Food Science, Faculty of Pharmacy, Complutense University of Madrid, Plaza Ramón y Cajal s/n, 28040 Madrid, Spain; esther.cuadrado@ucm.es (E.C.-S.); mlbermej@ucm.es (L.M.B.); araparic@ucm.es (A.A.); rortega@ucm.es (R.M.O.); 2 Nutritional Genomics and Epigenomics Group, Madrid Institute for Advanced Studies (IMDEA) Food Institute, Campus of International Excellence UAM+CSIC, 28049 Madrid, Spain; 3 UCM Research Group VALORNUT-920030, Complutense University of Madrid, Plaza Ramón y Cajal S/N, 28040 Madrid, Spain; aisabel.jimenezo@salud.madrid.org; 4 Pediatric Gastroenterology Unit, San Rafael Hospital, 28016 Madrid, Spain

**Keywords:** ENALIA study, breakfast, calcium, diet quality, pediatric nutrition, food sources, Spanish children, dairy products

## Abstract

The association between breakfast quality and total diet quality of children and adolescents ((1–17.9 years (*n* = 1570)) from the National Dietary Survey on the Child and Adolescent Population in Spain (ENALIA) was analyzed. Dietary information was collected using two non–consecutive one–day food diaries (1–10 years old) or two 24 h dietary recalls (>10 years). Breakfast quality index (BQI) and a variant of Nutrient Rich Foods index (NRF9.3) were calculated to assess the total diet quality. Children and adolescents who had breakfast on at least one day (*n* = 1561) were divided into two groups according to BQI: Worse Quality Breakfast (WQB) (BQI < 4 points (P66), *n* = 781) and Good Quality Breakfast (GQB) (BQI ≥ 4, *n* = 780). Younger children and those whose parents have university education presented higher BQI. GQB group had significantly higher intakes of micronutrients (vitamins A, D, C, B_1_, B_2_, B_6_, niacin, folate, calcium, potassium, magnesium). Fewer GQB children exceeded the Acceptable Macronutrient Distribution Range for fat and had folate and calcium intakes below their estimated average requirement. Daily NRF9.3 was 496.2 ± 54.0, being higher in GQB (503.8 ± 50.6 vs. 488.6 ± 56.2, *p* < 0.001). Increasing the quality of breakfast increased the possibility of having a NRF9.3 higher than P_50_ (OR: 1.893, CI: 1.549–2.315, *p* < 0.0001). Breakfasts have room for quality improvement in a high percentage of children. A higher quality breakfast is associated with a benefit in the quality of the total diet.

## 1. Introduction

Breakfast is part of a healthy diet and lifestyle and can have a positive impact on the health and well–being of children and adolescents [1,2,3,4,5]. This meal is considered by many as one of the most important of the day [6,7], as it breaks the fast maintained since the last intake of the previous day and modifies metabolic processes [8]: during fasting, glycogen reserves decrease and lipolysis increases, while with breakfast the use of glucose as an energy substrate increases and lipogenesis is also promoted. Children, compared to adults, have less capacity to adapt to the fasting situation than adults do [2,7,9] and have a greater metabolic stress induced by an overnight fast than adults have. Children have a higher brain glucose metabolism than adults [10]. They also have a relatively less muscle mass, which limits the availability of glycogenic amino acids. On the other hand, the longer duration of sleep, typical of childhood and adolescence, lengthens the night fast and can deplete glycogen stores during the night [11]. Thus, having or skipping breakfast, and the type of breakfast, could have a greater impact on children and adolescents than in adults. 

One of the benefits that has been associated with the habit of eating breakfast in children and adolescents is its protective effect against overweight and obesity [1,2,3,6,7,12,13,14,15] and its beneficial effect on cardiometabolic health indicators [7,14,15,16]. It has also been described that breakfast is associated with functional, cognitive and mood benefits [3,5,7,17]. This effect appears to be more evident in children whose nutritional status is compromised [7].

Despite the physical and mental benefits associated with eating breakfast, several representative national surveys in Europe and North America have shown that skipping breakfast is quite common [5,7,17,18,19]. Several authors have pointed out that between 10–30% of children skip breakfast [5,17]; a trend that increases in adolescence, especially in girls [15,18,20,21]. But much higher is the percentage of children who develop an inadequate/unbalanced breakfast pattern, considering the calories provided and the foods included in this meal [7,14,22,23].

Some studies indicate that a good–quality breakfast is recommended as part of a healthy diet because it is associated with a more adequate intake of macro– and micronutrients and a healthier lifestyle [7,14,24,25], although there is not enough evidence from longitudinal designs to clearly support the causal association between having breakfast and better health outcomes. Breakfast could reduce snacking and consumption of energy–rich, nutrient–poor foods throughout the rest of the day [5,12].

Breakfast consumption compared to skipping breakfast has been associated with better nutrient intake in various studies [25,26,27,28,29]. In fact, it is possible that the intake of various micronutrients relevant from a public health point [30], which have a key role in the growth and healthy development of children, may be affected by skipping breakfast. In addition, some of the foods consumed by children at breakfast, such as dairy products, breakfast cereals, and fruits, are important sources of micronutrients for this age group [12,13,27,31,32], although the role of breakfast cereals is also controversial because of the high content of added sugars or even salt and low content in fiber [33,34,35]. Moreover, breakfast habits in childhood track into adulthood, so they should be monitored and improved [6]. 

On the other hand, we must also consider that most of the research focuses on analyzing the problem of children who do not have breakfast [4,5,25,26], but it is also important to pay attention to the quality of breakfasts eaten [1,5,7,36]. In this sense, Matthys et al. [24] point out that consumers of a good–quality breakfast have higher quality diets (considering food consumption and nutrient intake) than consumers of a worse quality breakfast.

However, studies carried out on breakfast habits in representative samples of Spanish children are scarce [6,22,31,37], and the most recent studies uses samples either 6 years and older, 9 years and older or adolescents, respectively [6,22,37]. Some studies focus on special groups, such as groups of overweight/obese children [1] or specific Spanish populations [36], but there are few recent studies that were also carried out using representative samples of the population and very few have studied habits in children under 4 years of age.

It should also be taken into account that awareness campaigns on the importance of breakfast [23,37,38,39] may have changed habits in this first meal of the day, so having up–to–date data has become a topic of interest.

According to the above, the objective of this study was to analyze the breakfast habits and quality of a representative sample of Spanish children over one year of age and adolescents participating in the ENALIA study and to analyze the association of breakfast quality with children’s daily energy and nutrient intake and on the quality of their diet. We hypothesized that children and adolescents with better quality breakfasts also have better overall diet quality and nutrient intake.

## 2. Materials and Methods 

### 2.1. Study Design

The Encuesta Nacional de ALimentación en la Población Infantil y Adolescente de España (“National Dietary Survey on Spanish Children and Adolescents” or ENALIA) study is a cross–sectional study of a representative sample of Spanish children and adolescents (aged 6 months to 18 years). The ENALIA study was designed to collect food consumption data and information on eating habits in order to understand and monitor the diet of Spanish children and adolescents and to provide current data as a reference to analyze dietary trends over time. The design, protocol and methodology of the ENALIA study have already been published [40,41,42] and were made in accordance with the recommendations of the European Food Safety Agency (EFSA) “EU Menu” guidance recommendations [43,44]. 

The design and development of the ENALIA study was carried out by the Spanish Agency for Food Safety and Nutrition (by its Spanish acronym AESAN, formerly the Spanish Agency for Consumption, Food Safety and Nutrition, AECOSAN) with the aim of knowing and monitoring the diet of Spanish children and adolescents and to provide baseline information for the analysis of dietary trends over time [40].

The study was conducted in accordance with the guidelines set out in the Helsinki Declaration. Taking into account the age of the participants, information was provided to parents, guardians or other legal representatives, and the consent and approval of each participant was obtained before starting the interviews. The Spanish Agency for Food Safety and Nutrition (AESAN), belonging to the Spanish Ministry of Health, Consumption and Social Welfare, approved the study, which is considered appropriate from an ethical point of view, taking into account the Spanish Ethical Review System, given that the study to be performed did not imply any interventions, nor the collection of biological samples and all data were anonymous and de–identified [40].

### 2.2. Samples

Subjects were chosen randomly from the 17 autonomous regions of Spain, according to a random multistage sampling procedure, considering the census section and municipality, population size (<10,000, 10,000–100,000, 100,000–500,000, >500,000 inhabitants), sex and age. To obtain a representation of the intake in the different seasons, the subjects of each group were distributed uniformly across the four seasons. The study was also planned to control for an adequate proportion of weekdays and weekends and to represent homogeneously the different weeks of each month, for each group [40]. 

For each participant, five contacts were made: an initial contact by telephone, with the house or nurseries/kindergarten (in the case of children under 3 years), the second contact was made by letter (sending details of the study and consent to participate, as well as a general questionnaire and a diary/24 h recall), two other contacts were made by telephone (the third to confirm consent and administer the general questionnaire and the fourth to complete the first diary record/24 h recall), the last contact was made at the child’s home, to complete the second diary/24 h recall and perform the anthropometric study, as described in greater detail in previous publications [14,40,42].

The final sample of the ENALIA study included 1862 children aged 6 months to 17.9 years. In this study, we have considered the data of children between 1 and 17.9 years of age eating breakfast at least one of the two days of the study (*n* = 1561). 

### 2.3. Dietary Study

The dietary study was performed using two different methodologies depending on the child’s age: two non-consecutive one-day food diaries for children from six months to 10 years of age, or two 24 h dietary recalls for adolescents (11 years old and over). In both groups of children, the two one-day food diaries/24 h dietary recalls were separated by at least 14 days, trying to ensure that the information represents as much as possible the usual dietary intake. The interview schedule was organized to capture an adequate proportion of weekdays and weekend days at the population group level [40,41,42]. In this study sample (*n* = 1561), 72.8% of the dietary surveys correspond to weekdays and 27.2% to weekend days. Some participants provided information for two working days (46.1%), some for one working day and one weekend (46.4%) and a few (2.9%) for two different weekend days. 

For children aged 1 to 10 years, parents and caregivers completed the food diaries and the other questionnaires, consulting if necessary, with other proxy persons about the child’s diet out-or-home, such as the school canteen staff. For food diaries, parents (or caregivers) had to write down the food consumed and described the quantities using the most convenient system for them, either by weighing the quantities, or by describing them in natural units, by household measures or by using the photographic atlas [42]. For adolescents, they themselves were the respondents, but parents or caregivers were also present during interviews in order to provide details about the meals prepared at home.

In addition, a food propensity questionnaire (FPQ) specially designed for children and adolescents to complement the previous method was also applied once to each participant [44]. This questionnaire raised questions about the consumption of specific foods that may have a significant nutritional value or provide risk components [42].

The interviews were conducted by trained nutritionists/dieticians, using a computer with a specific software called ENIA–soft (version 5.0, Demométrica SL, Madrid, Spain). This software has the ability to collect not only dietary data from the 24-h recall/records, but also from the FPQ, and other data (sociodemographic, anthropometric and physical activity). The use of this software during the interview helped in the collection of data and facilitated the collection of information more homogeneously. The software used included a database with standard recipes, a database with coded foods, another with weights and household measurements, and another database with pictures and portion weights from a validated photographic food atlas for Spanish children and adolescents [42].

The images were used for the correct identification of the dishes, which were then broken down into their ingredients according to standard recipes. The quantities consumed were estimated using images supplemented with household measurements and portions indicated in standard recipes. For both food records and 24 h recalls, participants were asked about the use of salt on each occasion of eating (during preparation, cooking and on the table), and the type of salt consumed (regular or iodized) [40].

Nutrient intake was calculated using the Spanish Food Composition Tables [45] supplemented by additional nutrient content data for some branded foods or for enriched/fortified foods. The added sugar content was estimated using the flow chart described by Sluik et al. [46]. which is based upon the criteria of the International Scientific Committee of Choices [47].

### 2.4. Assessment of Dietary Quality

The Dietary Quality was analyzed using the Nutrient Rich Foods (NRF9.3) index [48,49]. The NRF9.3 is calculated as the sum of percent of recommended daily values (DVs) of nine nutrients whose consumption should be encouraged (Nutrient Rich subscore: protein; fiber; vitamins A, C, and D; Ca; Mg; Fe; K) minus the sum of the percentage of maximum recommended values (MRV) for three nutrients whose consumption should be limited (nutrients to limit (LIM) subscore: saturated fat, Na, and added sugars). To calculate the NRF9.3 index, the nutrient intake calculated from the 24-h recalls/food records was used. The overall daily intake of each nutrient for each participant was adjusted for energy intake by the residuals method [50] and then expressed as a percentage of the recommended DVs.

The DVs were determined (for sex and age categories) based on the Dietary Reference Intakes of the Institute of Medicine’s (IOM) [51,52,53,54,55,56,57]. Percent of DVs for nutrients to encourage were truncated at 100. An upper limit of 10% of energy intake was used as a MRV for added sugar, according to the recommendation of the WHO [58], and also for saturated fat [59], while for sodium the Tolerable Upper Intake Level (UL) according sex and age was used as MRV [57]. The maximum possible score was 900 points and a higher NRF9.3 score indicated a better quality of the overall diet.

### 2.5. Breakfast Quality

Breakfast was defined as the first eating occasion involving a solid food or a beverage that occurred after waking [24]. To assess the quality of this meal and taking into account the information reported in the 24 h recall/food records, the tool “Breakfast Quality Index (BQI)” was used. This item was first proposed by by Monteagudo et al. [36] based on data collected with an FFQ. Subsequently, other authors [1,60] have proposed slight modifications of this index to adapt it to other groups in which dietary information have been collected with 24 h recalls or food recalls. This index is composed of 10 items that value food groups (4 items), and energy and nutrients (6 items) of public health concern. The compliance with each of these items scores with 1 point, while non–compliance with the item is valued with 0 points. As a consequence, the BQI score ranged from 0 (poorest breakfast quality) to 10 points (optimal breakfast quality). Since there is no established a BQI cut-off point from which to define a good quality breakfast, we have considered a Good quality breakfast (GQB) when the BQI score was equal or higher than 4 (66th Percentile) and the quality was low (Worse quality breakfast, WQB) if the score was lower than that cutoff point.

The food group items included in the original BQI are (i) cereals and derivatives, (ii) fruits and/or vegetables, and (iii) dairy products. These items scored 1 point if they were consumed at breakfast at least in one of the two 24 h recalls/food records. In addition, a fourth item considered if cereals, fruits, and dairy products are included at the same breakfast on at least one day. The energy and nutrient items included in the BQI are: (i) “to provide <5% of total daily energy from food rich in simple sugars”; (ii) “to consume olive oil”; (iii) “to have a ratio between monounsaturated fatty acids (MUFA) to saturated fatty acids (SFA) ratio (MUFA: SFA) > median”; (iv) “to provide 20–25% of total daily energy intake by breakfast”; (v) “to provide 200–300 mg of calcium” and (vi) “not to consume butter or margarine”.

In our study we have applied the BQI with two modifications. First, we have adopted the modification proposed by Arenanza et al. [1] scoring 1 point the item “to have a ratio between monounsaturated to saturated fatty acids ratio (MUFA: SFA) ≥ 2:1”; and secondly, and taking into account that our sample has a wide age range and that the Recommended Dietary Allowances (RDAs) are very different at each age, we have adopted the criterion of Pereira et al. [60], which positively score this item when it exceeds 20% of the RDA. The mean intake from the two 24 h recall/food records was considered for scoring energy and nutrient items, except for “to consume olive oil” (+1 point was scored if at least one of the two days was consumed), and “not to consume butter or margarine” (+1 was scored if neither day was consumed).

The nutrient intake at breakfast is presented in the form of observed intakes and it has also been calculated the contribution of breakfast to the coverage of recommended intakes.

### 2.6. Handling of Misreporting

Misreporting of energy intake for the ENALIA study has been previously reported [40,41].

Subjects were identified as plausible under– or over–reporters of energy intake, considering the relationship between their energy intake (EI) and Basal metabolic rate (BMR), which was estimated using the equation of Schofield [61]. Under–reporters were identified as those with EI/BMR ratios under 0.73–1.08, while over–reporters were identified by EI/BMR ratios above 2.29–2.88 depending on the subject’s age and sex. Although it is important to value misreporting, following the EFSA [44] recommendations, misreporters were not excluded from the study, because their exclusion could modify the results of the research.

### 2.7. Socio–Demographic and Anthropometric Information

A general questionnaire on socio–demographic factors was applied, asking about the date of birth of the participants, the place/country of origin of the participant and their parents, the academic level of the parents, and about the health status of the participant (to know if they have any disease, follow a special diet, or consume any medication) [40,41].

The participants’ anthropometric data were measured at home by trained interviewers and following standardized procedures [62]. A stadiometer was used to measure the height in subjects of two years or more, and an infantometer to measure the length of subjects of 12 to 24 months of age, while remaining in a lying position. A digital weight scale with an accuracy of 0.1 kg was used. Weight was measured in kg, and height and length were measured in centimeters. Body mass index (BMI) was calculated, and World Health Organization (WHO) reference standards [63,64] were used to calculate BMI–Z scores. BMI for age Z score was used to categorize children as “underweight” (Z–BMI/age < −2 standard deviation (SD)), “normal BMI” (Z–BMI/age −2 to +1 SD), “overweight” (Z–BMI/age > +1 SD to <+2 SD) and obese (Z–BMI/age > +2 SD). Detailed information on the anthropometric measurements of the study children has been previously described [41].

### 2.8. Statistical Analysis

Considering that the average intake of a small number of days of dietary record/recall does not adequately reflect the usual intake, in the assessment of the daily intake it is necessary to apply a statistical model to dietary data to eliminate the day–to–day variation in food consumption [65]. For this purpose, we have used the method developed by Nusser et al. [66], also known as the Iowa State University (ISU) method. The ISU method consists of three steps: (1) a transformation that maps intakes into a normal scale; (2) a step to estimate the parameters of usual intake by using a measurement error framework; and (3) a back–transformation step to return the estimated usual intakes to their original scale. The ISU method was implemented using the PC–SIDE software (version 1.0, Department of Statistics, Centre for Agricultural and Rural Development, Ames, IA, USA, 2003), which was designed for this purpose. PC–SIDE estimated the percentiles of usual nutrient intake distributions and also an individual usual intake for each participant. The interview day (day 1 or day 2), the day of the week, season, and a sample weighting factor (to weight the sample to known population demographic characteristics) were considered in the adjustment of dietary data, stratifying by sex and age group [40,41,66].

For total dietary intakes the usual daily intakes (from food and beverage sources only) of macronutrients and micronutrients were estimated, and then adjusted by energy using the residuals method of Willett [50]. In addition, the usual percentage of total energy intake from protein, fat, carbohydrate and sugars were estimated. The consumption of food groups at breakfast was also adjusted by energy. For the energy and nutrients provided by breakfast, the observed intakes are presented, since it was not possible to transform the observed intakes into a normal scale and therefore estimate the usual intake.

To assess nutrient adequacy, the Estimated Average Requirement (EAR) cut–off point method was used [53,67]. For those nutrients for which an EAR is not available, the percentage of children with intakes lower than adequate intakes (AI) was estimated [27,67]. For macronutrients, both absolute amounts (g/day) and amounts relative to total energy intake (%E) were compared to recommended reference values. Acceptable macronutrient distribution ranges (AMDR) were used to assess whether an individual has contributions that are outside the AMDR [53]. Lastly, the fat profile was also estimated. For nutrients with established Tolerable Upper Intake Levels (ULs), the proportion of children with usual intakes exceeding the UL were estimated [67].

Statistical analyses were performed using the statistical software package SPSS version 20.0 (SPSS, Chicago, IL, USA) for Windows. A *p*–value < 0.05 indicated statistical significance. The normality of the distribution of the studied data was checked using the Kolmogorov–Smirnov test. Differences between males and females were examined with the Student’s *t*–test or the Mann–Whitney U test, depending on whether or not the data were normally distributed. Differences by age group were established with the Kruskal–Wallis test. Categorical variables were compared using the χ^2^ test. A Spearman regression analysis has also been applied, and the ORs have been calculated to analyze the impact of various influences on breakfast BQI, and also to calculate the impact of BQI on dietary quality indicators. To analyze differences in intake of macronutrients and micronutrients, of children with different BQI at breakfast, usual intakes (from food and beverage sources only) adjusted by energy are shown, using the Willet residual method, and differentiating by sex. Dietary intakes of the participants were expressed as the means and standard deviations, medians, and 5th and 95th percentiles [40].

## 3. Results

The final sample of the ENALIA study included 1862 children (aged between six months to 17.9 years), details of stratified multistage cluster–sampling in ENALIA study and of the individuals eliminated in each phase of the process can be seen in López–Sobaler et al. [41]. The present study analyzes the data of 1570 children from the ENALIA study, aged 1 to 17.9 years, considering that children aged 6–12 months may have a less structured breakfast, which could alter the results of the general analysis. In this article that analyzes the quality of the total diet according to the quality of breakfast we have focused especially on the 1561 children who ate breakfast, at least one of the two controlled days. In the group studied, 93.4% of children ate breakfast on the two controlled days, 0.6% did not eat breakfast on either day or 6% had breakfast on only one of the two days.

### 3.1. Description of the Sample

Table 1 presents personal and anthropometric data of the children studied and the educational level of their parents, differentiating by age and sex. This table highlights a significantly higher weight and height and a higher EI in boys, when compared to girls. The percentage of possible under–reporters (9.5%) was higher in children aged 11–17 (21.9%) than in children aged 1–10 years (3%). On the other hand, possible overestimation (1.9%) was higher in younger children (2.9%) (1–10 years) compared to adolescents (0.0%). The percentage of plausible reporters was 88.6% and the data presented in this report have not been adjusted for misreporting. Nevertheless, to check whether misreporting skews findings, comparisons of food and nutrient intakes were repeated using only the plausible reporters.

### 3.2. Breakfast Habits

The most consumed foods at breakfast were dairy, cereals, juices, bakery and fruit. Baby foods were consumed especially in children aged 1–3 years and their consumption decreased with age (*r* = –0.316, *p* < 0.05). However the consumption of other foods at breakfast increased with age, specifically dairy (*r* = 0.148, *p* < 0.05), cereals (*r* = 0.359, *p* < 0.05), fruit (*r* = 0.230, *p* < 0.05), protein foods (*r* = 0.326, *p* < 0.05), added products (sugar, honey, powdered chocolate, jam...) (*r* = 0.283, *p* < 0.05), fat (*r* = 0.133, *p* < 0.05), bakery (*r* = 0.348, *p* < 0.05) and juices (*r* = 0.209, *p* < 0.05). When comparing between age groups (Kruskal Wallis test), it was found that the consumption of dairy, added products, fat and bakery as lower and that of baby food was higher for children aged 1–3 years compared to the other groups. The consumption of fruit and juices was higher in children aged 9–13 and 14–17 years compared to 1–3 and 4–8 years, the consumption of cereals was higher for children aged 9–13 years compared to 1–3 and 4–8 years, and the consumption of protein foods and other foods was higher in children aged 9–13 and 14–17 years, compared to 1–3 years. Considering differences in terms of sex (Mann–Whitney), it was observed that boys aged 14–17 had a higher consumption of dairy and a lower consumption of baby foods when comparing with girls (Table 2).

Considering the average breakfast of all sample studied (*n* = 1561), this meal accounted for 18.3% of total calories and included 2.19 ± 0.85 foods from different categories. 13.4% of the children ate less than 1 food group per day at breakfast. The most consumed food groups by these children were dairy (60.3%), added products (56%) and baby foods (42.1%). On the other hand, 86.6% took ≥2 food groups per day, and the groups foods most included in their breakfast were dairy (90.4%), added products (90.5%) and cereals (66.6%).

More than half of the children who ate breakfast (56.4%) took less than 20% of their daily calories at breakfast, 27.5% took more than 25% and only 16.1% took what is recommended (between 20 and 25% of energy at breakfast).

### 3.3. Breakfast Quality

The average quality of breakfast evaluated by the BQI was 4.29 ± 1.26 (Table 3). Dairy products were the most consumed foods at breakfast (89.6% of children), followed by cereals and derivates (62.3%), while 15.6% include fruits and/or vegetables, and only 8.4% took the combination of dairy with cereals and fruits for breakfast. 40.0% of children took food low–sugars foods and very few children had a MUFA/SFA ratio ≥ 2:1. In the group of 14–17 years, girls took less often food rich in simple sugars at breakfast and had more often the recommended energy intake at breakfast (20–25% of energy), while more boys took more than 20% RDA of calcium. Table 4 shows the differences in food group consumption (adjusted for total energy intake) between children in the GQB and WQB group. Children with better breakfasts ate more amount of all food groups except added products, bakery and juices. In addition, all BQI items were significantly higher in children with GQB (who had a BQI of 5.13 ± 1.05) than in those with WQB (with a BQI of 3.37 ± 0.68) (Table 5). Children with GQB consumed more energy at breakfast (18.7 ± 4.0%), higher amounts of calcium (330.4 ± 81.9 mg) and less amount of simple sugars (24.3 ± 8.6 g), compared to children with WQB (whose breakfast provided 18.0 ± 4.6% of energy, 314.8 ± 83.8 mg of calcium and 26.2 ± 8.4 g of simple sugars).

### 3.4. Differences in Personal and Anthropometric Data According to the Quality of Breakfast

Table 6 presents the personal and anthropometric data of the children grouped by the quality of the breakfast. It shows that the WQB is more frequent in children of 9–13 years. As age increased, the probability of having a GQB decreased: OR: 0.314, CI: 0.236–0.418, *p* < 0.001 in children aged 4 to 8 years, OR: 0.284, CI: 0.214–0.376, *p* < 0.001 in children 9 to 13 years old and OR: 0.541, CI: 0.394–0.744, *p* < 0.001 in children 14 to 17 years old, considering the group of 1–3 years as the reference group). Parents of children with higher quality breakfasts (GQB) had a higher educational level. Children whose parents had university studies were more likely to have high–quality breakfasts compared to those with only compulsory studies (OR: 1.955, CI: 1.389–2.752, *p* < 0.001 for father’s studies; OR: 1.814, IC: 1.226–2.685, *p* = 0.003 for mother’s studies). Children using the computer for more than 1 h on weekend had less often GQB (OR: 0.758, CI: 0.597–0.963, *p* < 0.001). This influence was not observed in those who spent the same time on weekdays, or in those who spend more than 2 h watching TV on both weekend and weekdays. There were no differences in BMI nor in the prevalence of overweight or obesity between GQB and WQB children. Lower energy intake is also observed in children of 14–17 years with WQB, but EI/BMR does not present significant differences according to the quality of breakfast.

### 3.5. Dietary Quality According to the Quality of Breakfast

The supply of nutrients provided by breakfast (Table S1) was significantly higher for GQB in relation to proteins (g and %E), PUFA, fiber, and all vitamins and minerals. On the other hand, the contribution of breakfast to the recommended intakes were higher for GBQ and exceeded the 20% (Table S2) in relation to proteins, carbohydrates, vitamins A, B_1_, B_2_, B_6_, B_12_ and C, niacin, calcium, iron, zinc, magnesium, selenium and iodine.

In boys, those with a GQB had higher usual intakes of protein (g and %E) and lower daily energy intake, total sugars (g) and PUFA than those with WQB. On the other hand, GQB girls had lower daily energy and fat intake (%E), but higher protein intake (in g and %E), compared to WQB girls. Regarding micronutrients, both boys and girls with GQB had significantly higher intakes of vitamins A, D, B_1_, B_2_, B_6_, C, niacin, folate, calcium, potassium and magnesium comparing to children with WQB. In addition, GQB girls had higher intakes of iodine than WQB girls (Table 7). Having a GQB was associated with a higher likelihood of having higher calcium intakes than recommended (OR: 1.833, 95% CI: 1.492–2.252) (boys: OR: 1.718, 95% CI: 1.283–2.301 and girls: OR: 1.905, 95% CI: 1.419–2.559).

The mean daily NRF9.3 score was 496.2 ± 54.0, and it was significantly higher in the WQB group than in the GQB group, both in the total sample (488.6 ± 56.2 and 503.8 ± 50.6 respectively, *p* < 0.001) and both sex groups (Table 7). Having a GQB increased the likelihood of having a NRF9.3 greater than P50 (OR: 1.893, CI: 1.549–2.315, *p* < 0.0001).

There were fewer GQB children who exceeded the AMDR for fat and with folate and calcium intakes below their EARs. In girls, there were a higher prevalence of protein intakes exceeding AMDR and not reaching EAR for folate in GQB compared to WQB, but lower prevalence of insufficient intakes of vitamin E, calcium, zinc, magnesium and iodine (Table 8). In boys, there are lower prevalence of insufficient intakes of folate and calcium but higher prevalence for magnesium in GQB compared to WQB boys.

None of the children studied had higher intakes than UL in relation to calcium, iron, iodine, and vitamins B_6_, C, E and D. But it is confirmed that 0.8% of those studied (0.7% and 0.9% depending on having a WQB or GQB, NS) had folate intake > UL, 1.3% (0.6% and 1.9% depending on having a WQB or GQB, *p* < 0.05) exceeded the UL for selenium, and in relation to zinc, 16.2% (10.9% and 21.5% depending on having a WQB or GQB, *p* < 0.001) exceeded the UL marked for this mineral.

Breakfast habits and association between breakfast quality and total diet quality is maintained when only plausible reporters are considered (Tables S3–S9).

## 4. Discussion

The present study, carried out in a representative sample of Spanish children and adolescents from 1 to 18 years of age, shows that breakfast have room for quality improvement in a high percentage of children, especially adolescents, and that its higher quality is associated with a higher intake of vitamins and minerals and with higher general quality of the diet, evaluated by the NRF9.3. Our study presents several novel aspects: First, there are hardly any studies that relate the quality of breakfast with the quality of the total diet in children and adolescents [1,5,7,24,36]. However, knowing these aspects in this group is important to carry out nutrition education policies that contribute to improving their eating habits and their possible repercussions on their future health. Second, our sample is a representative sample of Spanish children and adolescents, which includes children under 6 years of age, which is a group in which there are few studies on their breakfast habits. And finally, the design, protocol and methodology of the ENALIA study were made in accordance with the recommendations of the European Food Safety Agency (EFSA) “EU Menu” guidance recommendations [43,44]. The sample studied presents anthropometric data similar to those of other Spanish children of the same age [1,6,21,22,31,32,36,37]. As far as misreporting is concerned, our results coincide with those of Coulthard et al. [2] which indicated that under–reporting may have been more prevalent for the children in the 11–18 years age group, who provided their own food consumption, in contrast to children in the younger age group, whose diaries were completed by their parents or caregivers. The percentage of misreporting was similar to that found in other studies [2,32].

### 4.1. Breakfast Habits Description

Although the figures comparison must be done with caution due to the different forms used to define breakfast in each study, several representative national surveys in North America and Europe have shown that breakfast skipping is highly prevalent [12,15,19]. Lazzeri et al., [6] analyzed the breakfast habits of adolescents (11–15 years) from 31 countries between 2002 and 2010, and found that there had been a decrease in the daily consumption of breakfast in Spain in that period. Based on the results of this study and other similar ones, there have been numerous breakfast promotion campaigns in Spain in recent years [22,23,31,36,37,38] so it is expected a decrease in the tendency to skip breakfast. In fact, in ENALIA study there are only 0.6% of children aged 1–17 years who do not eat breakfast on controlled days.

In the studied group, 93.4% of children ate breakfast on the two recorded days. This percentage is very similar to that found by Aranceta et al. [31] who indicated that 91.2% of the boys and 92.2% of the girls studied usually had breakfast (defined as any intake of food or beverage between 6 a.m. and 10 a.m. during weekdays and between 6 a.m. and 11 a.m. for the weekends and holidays). Similarly, the ANIBES (“anthropometric data, macronutrients and micronutrients intake, practice of physical activity, socioeconomic data and lifestyles in Spain”) study defined breakfast as the first intake declared by the participant after overnight sleeping, and found that 93.4% of children aged 9–12 years and 80.1% of children aged 13–17 years reported breakfast on a regular basis, while 6.6% and 19.9% of children aged 9–12 and 13–17, respectively, reported having breakfast irregularly or not having breakfast [23,37]. Guevara et al. [21] evaluated the quality of the breakfast of a group of Spanish adolescents (11–18 years old) based on the weekly frequency with which a “full breakfast” was consumed (when in addition to a glass of milk or a piece of fruit, the respondent eats something else), and observed that 24.5% of adolescents had a poor breakfast (skipping it two or more days a week), and it was more frequent as adolescents are older. Finally, in the ALADINO (“Alimentación, Actividad Física, Desarrollo Infantil y Obesidad”) study (carried out in a representative sample of the Spanish school population aged 6 to 9 years) 68,3% of families indicated that their children ate more than a drink (milk, juice, infusions, …) for breakfast every day and only 2.2% indicate that children never eat breakfast [22]. In this sense, in our study, only 0.4% of children and adolescents only drank milk or a juice for breakfast.

Dietary recommendations in Spain suggest that a healthy and a nutrient density–adequate breakfast should contribute around 20–25% to the total daily energy intake [23,29,31]. The breakfast of the children studied contributed an average of 18.3% of the daily energy, with 56.4% of children who took less than 20% of calories at breakfast. In this sense, other studies found lower energy intakes of breakfast, for example Matthys et al. [24] found that the contribution of breakfast to daily energy intake was on average 15.7% in boys and 14.9% in girls, and in the ANIBES study children’s breakfast contributed 18% of the energy and the observed in adolescents contributed 18% and 17% in men and women, respectively [37].

We also found in the present research 27.5% of children who took more than 25% of energy, while Matthys et al. [24] point out that a restricted number of children (9.7% in boys and 5.7% in girls, respectively) had a breakfast energy contribution higher than 25%. However, taking a lot of calories at breakfast is not a guarantee of having made a good breakfast, as foods rich in energy and poor in nutrients may have been included [7,37], for this reason it is appropriate to take into account other aspects assessed in the BQI.

The breakfast most frequently consumed in Spain consists of a dairy product, particularly skimmed or semi–skimmed milk and cereal, especially bread, which may differ from the findings in other European populations [3,14,23]). In the ALADINO study [22] the most common breakfast was constituted by a dairy plus a food of the cereal group (biscuits, breakfast cereals or bread/toast) (24.9%) or by dairy with pastries (30.3%). In the present study, these were the most frequent breakfasts also (dairy + cereal: 70.9% and dairy + bakery: 35.7%). As found in other studies dairy products were the most consumed foods at breakfast, followed by cereals and highlighting the low consumption of fruits and/or vegetables [22,23]).

### 4.2. Breakfast Quality

Among the criteria to define a quality and healthy breakfast, in addition to the regularity of the habit and its energy contribution, its nutritional quality should be considered [7]. The quality index used in the evaluation of breakfasts (BQI) [1] not only takes into account the amount of food consumed or the energy provided by breakfast, but also considers other items, such as the inclusion or not of some foods in the breakfast or the intake of critical nutrients.

The nutrient intake found at breakfast was somewhat lower than that found by Drewnowski et al. [68] and by Bellisle et al. [69] regarding fiber, vitamin B_6_, folate, vitamin C, iron, potassium and magnesium. But it was similar, or somewhat superior, to that reviewed in other studies [25,37]. This supply of nutrients received at breakfast is especially important in relation to nutrients that are ingested in insufficient quantity by a high percentage of individuals [30,32,40]. In the present study the breakfast provided more than 20% of the recommended intakes for proteins, vitamin A, B_1,_ B_2,_ B_6_, B_12_, C, niacin, folate, calcium, iron, zinc, magnesium, selenium and iodine (Table S2) but the breakfast was low in dietary fibre, and vitamins E and D, similarly to that observed in other studies [37].

Various studies indicate that breakfast should include foods at least from three key food groups, namely: starchy foods (cereals, pasta, bread), fruit and vegetables and milk and dairy products [23,29,31]. In this sense, studies carried out in the Spanish child population found that breakfast at these ages should be improved. For example, the ALADINO study [22] showed that breakfasts that included foods from the three recommended groups are scarce (only 2.2% of schoolchildren). In the present study the consumption of the three types of food is higher (8.4%) but is still insufficient.

Taking into account that the mean BQI in our study is 4.25 and that the maximum score is 10, the possibility of improvement is highlighted. There are aspects of breakfast that can be improved in the whole group, but specifically children whose breakfast has a BQI < 4 (50% of the total) have the most room for improvement of their breakfast. The key aspects that could improve these children and adolescents of the WQB group are the inclusion of fruit/vegetables in breakfast, including cereals, fruits and dairy in the same meal, and the preferential use of MUFA–rich fats (olive oil, vegetable oil) at breakfast, reducing the consumption of bakery and juices.

### 4.3. Association between the Quality of Breakfast and Dietary Quality

Several studies have indicated that the breakfast habit is associated with more correct and balanced eating habits in both children and adolescents [2,4,12,20,24,25,26,27,28,31]. However, fewer studies relate the quality of breakfast to the quality of the total diet and the potential risk of having insufficient or excessive intakes [27].

Regarding the impact of breakfast on macronutrient intake, several studies indicate that both children who skip breakfast and those who have poorer quality breakfasts consume more fat, saturated fat and cholesterol and less carbohydrates and fiber [2,4,5,12,13,28]. Although results from different studies are not conclusive, most of them focus on skipping breakfast but not on its quality [24]. In our study, and similarly to Afeiche et al. [26], Matthys et al. [24] and Sjoberg et al. [20], children with GQB had a higher intake of protein (g and %E) than WQB group, while GQB boys had lower intake of total sugars (g) and GQB girls had lower relative intake of total fat (%E) (Table 7). Coinciding with the results of other studies, in both boys and girls the intake of most micronutrients was significantly higher in GQB (Table 7). Specifically, in children who take a higher quality breakfast various authors found higher intake of folates [12,13,28,31], calcium and magnesium [12,13,20,24,27,28,31], vitamins A [12,13] and D [26,27], vitamins B_1_ and B_2_ [12,13,20,24,28], vitamin B_6_ and niacin [12,26], potassium [12,13,28], iodine [13] and vitamin C [20,25].

These results highlight the importance of a quality breakfast, since the intake of nutrients such as calcium, magnesium and vitamin D is insufficient in a high percentage of the population [32,40,70] and in the group studied in particular (Table 8). In addition, the presence of dairy products in this meal seems important, since they are the main source of these nutrients in the Spanish diet [7,32,71]). Specifically the contribution of calcium is particularly important because is a vital nutrient for bone growth and needs in childhood are high due to rapid growth and bone mass accretion and given that its contribution is insufficient in a high percentage of Spanish children [32,40]. Dairy products can also provide SFA, especially whole–fat dairy products. In general, reducing the intake of SFA is recommended [72], despite the fact that there is growing evidence that questions this recommendation [73]. In particular, dairy products appear to have a beneficial effect on cardiometabolic risk factors, compared to other animal sources of SFA [74]. On the other hand, dairy products are susceptible to incorporating added sugars in their composition, so it is advisable to include them in the diet as part of a main meal and in their most natural form, such as sugar–free dairy products [75].

Adolescents who have a WQB seem unable to compensate for low micronutrient intake at other meals throughout the day. In a recent review of Giménez–Legarre et al. [4], all of the above findings are reflected and it is clear that there is evidence that breakfast consumption contributes significantly to the overall nutrient adequacy of the diet [5,24]. In fact, the breakfasts studied cover a significant percentage of the recommended intakes, especially among people with GQB (Table S2).

Regarding the possible risk of excess, we observed a low percentage of children who exceeded the UL for folate and selenium, but a considerable percentage who exceed the UL for zinc, higher among those with a GQB. Some authors such as Barr et al. [27] point out that breakfast consumption would be associated with a lower prevalence of nutrient inadequacy as assessed using the DRI framework, and would have little or no impact on the potential risk of excessive nutrient intakes. We agree with the comments of Barr et al. [27], but consider that the insufficient and excessive contribution of zinc should be the subject of future studies.

### 4.4. Strengths and Limitations

One of the major strengths of the present study is the large representative sample size of the Spanish children aged one to <18 years. The proportioned selection sample (by age, sex, geographical areas, population size) provides representative data about Spanish children. The study has also been designed to get a representation of the intake in the different seasons, with an adequate proportion of weekdays and weekends [40]. Furthermore, standardized and validated procedures have been used, following the recommendations of the European Food Safety Agency (EFSA) “EU Menu” guidance recommendations [43,44], what also allows comparisons with data from other countries following the same methodology.

The 24 h dietary record in children and 24 h dietary recalls in adolescents are appropriate methods for getting information about the types and amounts of food consumed [43,44,76,77]. Also, the data of the group of children aged 9–13 years should be evaluated with caution since in this age group the dietary data were collected using two different methodologies (24 h recall for children up to 10 years old and food record for children 11 and older). Application of the ISU method corrected the data for day–to–day variation, although we should still bear in mind that the true intake distributions remain unknown because of the lack of objective validation data [40].

One of the limitations of the study is that the food composition tables may not accurately reflect the nutrient composition of the food. There may be differences in the composition of foods, depending on their origin, season, and mode of cultivation or variety. Moreover, Spanish Food Composition Tables used include the composition of various enriched/fortified foods most frequently available in Spain, and additional composition data for specific brands were taken into account. But the composition of these foods can change between populations or depending on the brand, if not known exactly. Our study did not account for the use of supplements of vitamins and minerals, so the results of this study are limited to the intake of micronutrients from the diet [40]. However, we know that the consumption of supplements is very low (the most frequent is vitamin D in 1.5% of children, and multivitamins/multivitamins and minerals in 1.4%) and has been described in a previous publication [40].

Our study had a cross–sectional design that highlighted the existence of associations but did not demonstrate a causal relationship. On the other hand, age differences make younger children less able to remember and cooperate in dietary assessment procedures than older children, so information should be obtained from parents or caregivers, who have no direct knowledge of the child’s consumption at school and outside the home. For these children they may be given under– or overreporting derived from parents own beliefs related to diet and health. Older children have a greater role in the realization of dietetic study, but there may also be trends to overestimate foods accepted as healthy and underestimate the least healthy options. To address this last limitation the misreporting of the ENALIA participants was previously assessed and reported by López–Sobaler et al. [41]. In these children and adolescents who ate breakfast and are the object of the present paper, the percentage of plausible reporters is 88.6%. The data presented in this paper have not been adjusted for misreporting, although it has been found that the association between breakfast quality and total diet quality is maintained when only plausible reporters are considered.

Finally, the comparison of our results with those of other studies is limited by the differences in the definition of breakfast, and the differences in the methods used to evaluate the diet.

## 5. Conclusions

The present study provides information on the breakfast habits of Spanish children aged 1–17.9 years and highlights the association between higher quality breakfasts with higher diet quality, and endorses the importance of improving the first meal of the day as an important element of a healthy dietary pattern in children. A high percentage of children, especially adolescents, have room for improve their breakfast. It is advisable to avoid suppressing it and improve its quality, increasing the consumption of cereals (especially high–fiber and nutrient–rich whole grains), fruits, and dairy products, trying to combine them in the same meal, choosing preferably MUFA –rich fats and limiting the consumption of bakery and juices. It is recommended to improve policies and interventions by health professionals that support good quality breakfast consumption, as this simple message could help improve adherence to nutritional recommendations.

## Figures and Tables

**Table 1:** Personal and anthropometric characteristics of children aged 1 to 17.9 years studied in the ENALIA Study, 2013–2014 (National Dietary Survey on the child and Adolescent Population). Details of children who take breakfast.

	Total	Boys	Girls
N	1561	824	737
Age (years) X ± SD	8.0 ± 5.0	8.1 ± 5.0	7.9 ± 4.9
Age group. *n* (%)			
1–3 years	406 (26)	217 (26.3)	189 (25.6)
4–8 years	416 (26.6)	210 (25.5)	206 (28)
9–13 years	468 (30)	242 (29.4)	226 (30.7)
14–17 years	271 (17.4)	155 (18.8)	116 (15.7)
Anthropometric characteristics			
Weight (kg) X ± SD #	33.2 ± 18.3	34.4 ± 19.4	31.9 ± 17 **
Height (cm) X ± SD #	129.7 ± 30.6	131.3 ± 31.7	127.9 ± 29.2 *
BMI (kg/m^2^) X ± SD #	17.9 ± 3.1	18.0 ± 3.0	17.8 ± 3.1
Z–BMI #	0.32 ± 1.22	0.31 ± 1.22	0.33 ± 1.21
Father’s highest educational level. *n* (%)			
Mandatory or less	179 (11.6)	106 (13.1)	73 (10.0)
Secondary	438 (28.4)	221 (27.3)	217 (28.8)
Vocational training/Bachelors	323 (21)	176 (21.7)	147 (20.2)
University	600 (39)	308 (38.0)	292 (40.1)
Mother’s highest educational level. *n* (%)			
Mandatory or less	121 (7.8)	72 (8.8)	49 (6.7)
Secondary	377 (24.2)	192 (23.4)	185 (25.2)
Vocational training/Bachelors	319 (20.5)	167 (20.3)	152 (20.7)
University	739 (47.5)	390 (47.5)	349 (47.5)
Energy usual intake (EI) (kcal/day) #			
1–3 years	1451 ± 202	1497 ± 210.8	1398 ± 177.6 **
4–8 years	1780 ± 214.5	1880 ± 214.6	1679 ± 159.9 **
9–13 years	2024 ± 258.7	2137 ± 229.3	1903 ± 232.5 **
14–17 years	2199 ± 440.2	2412 ± 415.5	1915 ± 285.4 **
EI/BMR #			
1–3 years	2.05 ± 0.37	2.04 ± 0.37	2.07 ± 0.37
4–8 years	1.75 ± 0.25	1.77 ± 0.25	1.72 ± 0.25
9–13 years	1.53 ± 0.26	1.53 ± 0.25	1.52 ± 0.26
14–17 years	1.37 ± 0.25	1.38 ± 0.27	1.35 ± 0.23

Significant differences between sex groups are shown (* *p* < 0.05, ** *p* < 0.001), applying the chi–square and Mann–Whitney tests. # Variable does not follow a normal distribution. SD: standard deviation; BMI: body mass index. Z–BMI: z–score for BMI. EI: Energy intake, BMR: basal metabolic rate.

**Table 2:** Breakfast food consumption by age group (g/day adjusted by energy).

		1–3 Years	4–8 Years	9–13 Years	14–17 Years	*p*-Value †	14–17 Years		*p*-Value $
							Boys	Girls	
N		406	416	468	271		155	116	
Dairy	X ± SD	142.8 ± 121.2 ^a^	201.8 ± 74.2 ^b^	197.9 ± 83.2 ^b^	191.1 ± 95.0 ^b^	<0.001	206.0 ± 93.5	172.7 ± 94.0	0.010
	P50 (P5–P95)	151.4 (0.0–305.0)	216.1 (5.0–294.8)	207.0 (0.0–320.0)	204.8 (0.0–340.3)		212.1 (0.0–351.3)	200.8 (0.0–302.0)	
Cereals	X ± SD	15.6 ± 30.7 ^a^	18.4 ± 26.8 ^a^	23.1 ± 29.5 ^b^	20.4 ± 33.5 ^a,b^	<0.001	19.8 ± 25.7	21.3 ± 41.1	0.902
	P50 (P5–P95)	5.8 (0.0–50.3)	11.8 (0.0–56.4)	16.9 (0.0–71.9)	10.5 (0.0–72.3)		11.8 (0.0–72.6)	9.8 (0.0–71.6)	
Fruits	X ± SD	6.7 ± 37.0 ^a^	9.3 ± 40.8 ^a^	19.8 ± 80.3 ^b^	28.1 ± 83.3 ^b^	<0.001	24.9 ± 79.8	32.0 ± 87.4	0.454
	P50 (P5–P95)	0.0 (0.0–40.1)	0.0 (0.0–72.6)	0.0 (0.0–126.0)	0.0 (0.0–148.6)		0.0 (0.0–148.6)	0.0 (0.0–175.3)	
Protein foods	X ± SD	0.7 ± 3.8 ^a^	1.8 ± 7.9 ^a,b^	2.2 ± 7.1 ^b^	3.2 ± 14.3 ^b^	0.001	3.8 ± 17.9	2.4 ± 8.0	0.86
	P50 (P5–P95)	0.0 (0.0–3.2)	0.0 (0.0–12.2)	0.0 (0.0–16.5)	0.0 (0.0–23.0)		0.0 (0.0–25.4)	0.0 (0.0–19.5)	
Added Products	X ± SD	3.1 ± 5.6 ^a^	6.8 ± 8.4 ^b^	7.2 ± 9.8 ^b^	7.5 ± 7.3 ^b^	<0.001	7.0 ± 6.7	8.1 ± 8.0	0.398
	P50 (P5–P95)	0.0 (0.0–13.8)	5.4 (0.0–19.6)	5.1 (0.0–19.1)	6.3 (0.0–22.3)		6.2 (0.0–21.3)	6.3 (0.0–26.8)	
Fats and oils	X ± SD	0.5 ± 1.9 ^a^	1.8 ± 3.6 ^b^	2.0 ± 3.9 ^b^	1.7 ± 3.7 ^b^	<0.001	1.8 ± 3.6	1.6 ± 4.0	0.485
	P50 (P5–P95)	0.0 (0.0–4.3)	0.0 (0.0–9.8)	0.0 (0.0–9.8)	0.0 (0.0–9.1)		0.0 (0.0–9.2)	0.0 (0.0–8.7)	
Baby foods	X ± SD	69.4 ± 106.5 ^a^	4.8 ± 31.6 ^b^	0.7 ± 10.3 ^b^	0.5 ± 6.9 ^b^	<0.001	0.0 ± 0.0	1.2 ± 10.3	0.044
	P50 (P5–P95)	9.7 (0.0–290.3)	0.0 (0.0–4.4)	0.0 (0.0–0.0)	0.0 (0.0–0.0)		0.0 (0.0–0.0)	0.0 (0.0–0.0)	
Bakery	X ± SD	13.5 ± 28.5 ^a^	21.9 ± 33.8 ^b^	21.1 ± 31.6 ^b^	21.7 ± 30.5 ^b^	<0.001	20.0 ± 25.7	23.8 ± 35.5	0.774
	P50 (P5–P95)	0.0 (0.0–61.2)	9.3 (0.0–87.0)	9.2 (0.0–77.3)	10.5 (0.0–76.3)		10.2 (0.0–72.1)	10.6 (0.0–98.3)	
Juices	X ± SD	8.2 ± 31.7 ^a^	15.6 ± 42.8 ^a^	23.5 ± 52.9 ^b^	27.0 ± 52.8 ^b^	<0.001	26.8 ± 52.9	27.2 ± 52.7	0.685
	P50 (P5–P95)	0.0 (0.0–64.0)	0.0 (0.0–104.3)	0.0 (0.0–140.8)	0.0 (0.0–154.2)		0.0 (0.0–176.3)	0.0 (0.0–151.9)	
Other foods	X ± SD	0.6 ± 2.9 ^a^	0.6 ± 2.9 ^a,b^	0.9 ± 3.6 ^b^	1.2 ± 4.7 ^b^	0.001	1.5 ± 5.5	0.8 ± 3.5	0.908
	P50 (P5–P95)	0.0 (0.0–4.6)	0.0 (0.0–4.3)	0.0 (0.0–8.5)	0.0 (0.0–9.0)		0.0 (0.0–10.9)	0.0 (0.0–3.0)	

No variable follow a normal distribution. † Significant differences (*p*-Value) between age groups applying Kruskal Wallis test. $ Significant differences (*p*-Value) between sex groups in adolescents (14–17 years old) applying Mann–Whitney U test. SD: standard deviation. Different superscripts (a, b) denotes significant difference between groups of age (*p* < 0.001). Added products: Other foods that can help make this food more appetizing (sugar, honey, powdered chocolate, jam…).

**Table 3:** Breakfast Quality Index (BQI) components and number of children meeting the criteria (*n*, %) by age group.

	Total Sample	1–3 Years	4–8 Years	9–13 Years	14–17 Years	*p*-Value †	14–17 Years		*p*-Value †
							Boys	Girls	
N	1561	406	416	468	271		155	116	
BQI Item, *n* (%)									
Cereals and derivate consumption	1094 (62.3)	103 (55.5) ^a^	92 (61.2) ^b,c^	409 (69.4) ^b^	195 (58.1) ^a,c^	<0.001	103 (55.5)	92 (61.2)	0.296
Fruits and/or vegetables consumption	274 (15.6)	39 (21.3) ^a^	36 (24.1) ^a^	109 (18.5) ^b^	76 (22.6) ^c^	<0.001	39 (21.3)	36 (24.1)	0.541
Dairy products consumption	1573 (89.6)	172 (92.9) ^a^	132 (87.9) ^b^	559 (95) ^b,c^	304 (90.7) ^c^	<0.001	172 (92.9)	132 (87.9)	0.118
Food rich in simple sugars (<5%E)	702 (40.0)	42 (22.6) ^a^	49 (32.8) ^b^	201 (34.1) ^c^	91 (27.1) ^c^	<0.001	42 (22.6)	49 (32.8)	0.041
Include MUFA–rich fats	243 (13.8)	26 (14.2) ^a^	17 (11.2) ^b^	100 (17) ^b^	43 (12.9) ^a,b^	<0.001	26 (14.2)	17 (11.2)	0.459
MUFA/SFA ratio (≥2:1)	19 (1.1)	4 (1.9) ^a^	3 (1.7) ^a^	6 (1.1) ^a^	6 (1.8) ^a^	0.531	4 (1.9)	3 (1.7)	0.911
Energy intake from breakfast (20–25%E)	287 (16.3)	31 (16.8) ^a^	34 (22.4) ^a^	112 (19) ^a^	65 (19.3) ^a^	<0.001	31 (16.8)	34 (22.4)	<0.001
Fruits. cereals and dairy product in the breakfast	148 (8.4)	21 (11.6) ^a^	19 (12.9) ^a,b^	62 (10.5) ^b,c^	41 (12.2) ^c^	<0.001	21 (11.6)	19 (12.9)	0.726
Calcium (>20% RDA)	1484 (84.5)	155 (83.9) ^a^	109 (72.4) ^a^	463 (78.7) ^b^	264 (78.7) ^b^	<0.001	155 (83.9)	109 (72.4)	0.013
Absence of butter or margarine	1701 (96.9)	174 (94.2) ^a^	145 (96.6) ^a,b^	566 (96.2) ^b^	319 (95.3) ^b,c^	<0.001	174 (94.2)	145 (96.6)	0.265
Breakfast quality BQI score (0–10), X ± SD	4.29 ± 1.26	4.03 ± 1.18 ^a^	4.37 ± 1.2 ^b^	4.39 ± 1.33 ^b^	4.19 ± 1.27 ^a,b^	<0.001	4.15 ± 1.28	4.23 ± 1.27	0.547

† The chi square test has been applied. Different superscripts (a,b,c) denotes significant differences between groups of age in the two-sided equality test for column proportions. RDA: Recommended Dietary Allowance (IOM, 2000).

**Table 4:** Breakfast food consumption by breakfast quality group (g/day adjusted by energy).

		WQB (BQI < P66)	GQB (BQI ≥ P66)	*p*-Value †
N		781	780	
Dairy	X ± SD	178.6 ± 97.7	200.0 ± 85.3	<0.001
	P50 (P5–P95)	203.5 (0.0–316.3)	214.4 (0.0–312.6)	
Cereals	X ± SD	14.4 ± 33.0	26.1 ± 24.5	<0.001
	P50 (P5–P95)	0.0 (0.0–59.4)	21.5 (0.0–71.6)	
Fruits	X ± SD	7.1 ± 36.4	25.8 ± 85.9	<0.001
	P50 (P5–P95)	0.0 (0.0–54.9)	0.0 (0.0–138.3)	
Protein foods	X ± SD	1.1 ± 5.8	3.1 ± 11.2	<0.001
	P50 (P5–P95)	0.0 (0.0–0.0)	0.0 (0.0–19.7)	
Added Products	X ± SD	7.3 ± 9.7	5.4 ± 6.7	<0.001
	P50 (P5–P95)	5.5 (0.0–21.3)	4.1 (0.0–15.9)	
Fats and oils	X ± SD	1.2 ± 3.6	2.1 ± 3.5	<0.001
	P50 (P5–P95)	0.0 (0.0–9.2)	0.0 (0.0–9.2)	
Baby foods	X ± SD	15.2 ± 61.7	11.0 ± 41.9	0.011
	P50 (P5–P95)	0.0 (0.0–118.6)	0.0 (0.0–88.5)	
Bakery	X ± SD	26.7 ± 35.2	13.1 ± 25.5	<0.001
	P50 (P5–P95)	15.5 (0.0–92.9)	0.0 (0.0–56.3)	
Juices	X ± SD	21.4 ± 50.8	16.8 ± 43.1	0.039
	P50 (P5–P95)	0.0 (0.0–147.9)	0.0 (0.0–102.7)	
Other foods	X ± SD	0.7 ± 3.2	0.9 ± 3.9	<0.001
	P50 (P5–P95)	0.0 (0.0–6.6)	0.0 (0.0–4.4)	

No variable follow a normal distribution. † Significant differences (*p*-value) between WQB with GQB groups applying Mann–Whitney test. SD: standard deviation. WQB: Worse quality breakfast, GQB: Good quality breakfast. Added products: Other foods that can help make this food more appetizing (sugar, honey, powdered chocolate, jam…).

**Table 5:** Breakfast Quality Index (BQI) components and number of children meeting the criteria (*n*, %) by quality breakfast group.

	**WQB (BQI < P66)**	**GQB (BQI ≥ P66)**	***p* Value †**
**N**	**781**	**780**	
BQI Item, *n* (%)			
Cereals and derivate consumption	381 (41.2)	713 (85.7)	<0.001
Fruits and/or vegetables consumption	55 (5.9)	219 (26.3)	<0.001
Dairy products consumption	791 (85.7)	782 (93.9)	<0.001
Food rich in simple sugars (<5%E)	261 (28.3)	441 (53)	<0.001
Include MUFA–rich fats	11 (1.2)	232 (27.8)	<0.001
MUFA/SFA ratio (≥2:1)	0 (0)	19 (2.3)	<0.001
Energy intake from breakfast (20–25%E)	71 (7.7)	216 (25.9)	<0.001
Fruits, cereals and dairy product in the breakfast	0 (0)	148 (17.8)	<0.001
Calcium (>20% RDA)	711 (77)	773 (92.9)	<0.001
Absence of butter or margarine	877 (95)	824 (99)	<0.001
Breakfast quality BQI score (0–10), X ± SD	3.42 ± 0.68	5.25 ± 1.05	<0.001

† The chi square test has been applied. RDA: Recommended Dietary Allowance (IOM, 2000). WQB: Worse quality breakfast, GQB: Good quality breakfast.

**Table 6:** Characteristics of children aged 1 to 17 y, by breakfast quality (*n* = 1561).

	Total Sample	WQB BQI < P66 (<4)	GQB BQI ≥ P66 (≥4)	*p*-Value †
N	1561	781	780	
Sex, *n* (%)				0.032
Male	903 (51.4)	452 (49)	450 (54.1)	
Female	853 (48.6)	471 (51)	382 (45.9)	
Age group. *n* (%)				<0.001
1–3 years	289 (16.4)	88 (9.6)	200 (24)	
4–8 years	543 (30.9)	320 (34.6)	223 (26.8)	
9–13 years	589 (33.5)	361 (39.1)	228 (27.4)	
14–17 years	335 (19.1)	154 (16.7)	181 (21.7)	
Community size. *n* (%)				0.702
<10,000	341 (19.4)	189 (20.5)	152 (18.3)	
10,000–100,000	728 (41.5)	380 (41.2)	348 (41.8)	
100,000–500,000	429 (24.4)	221 (23.9)	208 (25)	
>500,000	257 (14.7)	133 (14.4)	124 (14.9)	
Father’s highest educational level. *n* (%)				<0.001
Mandatory or less	210 (12.1)	137 (15)	74 (9)	
Secondary	497 (28.7)	265 (29.2)	232 (28.2)	
FP superior/Bachelors	361 (20.9)	191 (21.1)	170 (20.6)	
University	663 (38.3)	314 (34.7)	348 (42.3)	
Mother’s highest educational level. *n* (%)				0.003
Mandatory or less	146 (8.4)	91 (9.9)	55 (6.6)	
Secondary	434 (24.8)	239 (26)	195 (23.4)	
Vocational training/Bachelors	358 (20.4)	196 (21.4)	161 (19.4)	
University	812 (46.4)	391 (42.6)	421 (50.6)	
Sedentary free time. *n* (%)				
≥1 h using computer on weekdays	437 (29.9)	253 (31.3)	184 (28.3)	0.205
≥1 h using computer on weekend	898 (61.5)	521 (64.4)	377 (57.8)	0.009
≥2 h using TV on weekdays	365 (25.0)	210 (26.0)	154 (23.7)	0.298
≥2 h using TV on weekend	1034 (70.8)	579 (71.6)	455 (69.8)	0.434
Anthropometric characteristics. X ± SD				
Weight (kg) #	35.6 ± 17.6	35.9 ± 16.1	35.4 ± 19.1	0.018 $
Height (cm) #	134.8 ± 27.8	136.3 ± 24.7	133.1 ± 30.8	0.013 $
BMI (kg/m^2^) #	18.1 ± 3.1	18.1 ± 3.1	18.1 ± 3.1	0.233 $
Z–BMI/Age #	0.3 ± 1.2	0.3 ± 1.2	0.4 ± 1.2	0.091 $
Weight status by WHO. *n* (%)				0.474
Underweight	32 (1.9)	17 (2)	15 (1.9)	
Normal	1158 (69.8)	608 (69.7)	551 (69.8)	
Overweight	344 (20.7)	188 (21.6)	156 (19.8)	
Obese	126 (7.6)	58 (6.7)	67 (8.5)	
Energy intake (kcal/day) #				
1–3 years	1468 ± 209	1467 ± 204	1469 ± 211	0.776
4–8 years	1779 ± 214	1780 ± 218	1778 ± 209	0.815
9–13 years	2020 ± 258	2023 ± 259	2014 ± 257	0.856
14–17 years	2189 ± 439	2087 ± 382	2276 ± 465	<0.001
EI/BMR #				
1–3 years	2.02 ± 0.37	2.03 ± 0.37	2.01 ± 0.38	0.302
4–8 years	1.75 ± 0.25	1.76 ± 0.24	1.72 ± 0.26	0.079
9–13 years	1.53 ± 0.26	1.54 ± 0.25	1.5 ± 0.27	0.082
14–17 years	1.37 ± 0.25	1.35 ± 0.23	1.38 ± 0.27	0.372

† Significant differences (*p*–values) between WQB with GQB groups applying the chi–square, Mann–Whitney tests or ANCOVAs. # Variable did not follow a normal distribution. WQB: Worse quality breakfast, GQB: good quality breakfast. $ The ANCOVA test was applied adjusting for age.

**Table 7:** Usual intakes (from food and beverage sources only) adjusted by energy of macronutrients and micronutrients in Spanish children and adolescents by sex and type of breakfast (*n* = 1561).

	Boys					Girls				
	Worse Quality Breakfast (WQB)		Good Quality Breakfast (GQB)		*p* Value †	Worse Quality Breakfast (WQB)		Good Quality Breakfast (GQB)		*p* Value †
	Mean ± SD	Median (P5–P95)	Mean ± SD	Median (P5–P95)		Mean ± SD	Median (P5–P95)	Mean ± SD	Median (P5–P95)	
Energy (KJ/day) #	8.437 ± 1.417	8.492 (6.142–10.696)	8.390 ± 1.883	8.212 (5.547–11.667)	0.010	7.452 ± 1.105	7.418 (5.680–9.331)	7.217 ± 1.249	7.114 (5.266–9.435)	<0.001
Energy (kcal/day) #	2.017 ± 339	2.030 (1.468–2.556)	2.005 ± 450	1.963 (1.326–2.788)	0.010	1.781 ± 264	1.773 (1.358–2.230)	1.725 ± 299	1.700 (1.259–2.255)	<0.001
Protein g/day#	76.1 ± 7.9	75.1 (65.2–88.9)	77.2 ± 8.1	77.1 (64.1–92.0)	0.028	75.5 ± 8.1	74.9 (62.5–90.0)	77.1 ± 8.0	76.1 (65.3–92.4)	0.005
Protein (%E) #	16.8 ± 1.7	16.6 (14.3–19.7)	17.0 ± 1.8	17.0 (14.2–20.3)	0.017	16.7 ± 1.8	16.6 (13.9–19.9)	17.1 ± 1.8	16.8 (14.6–20.7)	0.002
Carbohydrates, total (g/day)	212.7 ± 15.4	212.4 (188.6–238.7)	212.0 ± 18.7	211.6 (180.6–241.9)	0.546	210.8 ± 17.2	211.8 (179.8–238.8)	211.1 ± 15.8	210.3 (185.7–237.0)	0.799
Carbohydrates, total (%E)	46.9 ± 3.4	46.9 (41.7–52.6)	46.8 ± 4.1	46.6 (40.0–53.3)	0.573	46.6 ± 3.8	46.8 (39.8–52.8)	46.7 ± 3.5	46.4 (41.1–52.4)	0.736
Total sugars (g/day)	100.4 ± 16.9	100.2 (69.9–128.3)	97.9 ± 16.1	97.5 (71.1–124.6)	0.022	92.7 ± 16.8	92.6 (65.4–122.2)	93.3 ± 14.9	93.3 (68.1–116.9)	0.598
Total sugars (%E)	21.5 ± 3.9	21.5 (14.9–28.0)	21.2 ± 4.0	21.2 (14.9–27.8)	0.155	20.8 ± 4.0	20.7 (14.2–27.3)	21.2 ± 3.7	21.2 (15.4–27.2)	0.115
Fat, total (g/day) #	69.2 ± 6.4	69.7 (58.0–79.0)	69.0 ± 6.8	69.1 (57.3–81.1)	0.726	70.0 ± 6.5	69.7 (59.8–80.2)	68.7 ± 6.7	69.1 (56.2–78.7)	0.057
Fat, total (%E) #	34.6 ± 3.2	34.8 (29.0–39.4)	34.5 ± 3.4	34.5 (28.7–40.6)	0.670	34.9 ± 3.2	34.8 (29.7–40.1)	34.3 ± 3.3	34.5 (28.1–39.3)	0.046
SFA (g/day) #	24.5 ± 3.8	24.8 (18.0–30.3)	24.5 ± 4.3	24.4 (17.8–32.1)	0.464	25.1 ± 4.0	25.3 (18.3–31.0)	24.9 ± 4.8	24.7 (18.0–33.1)	0.863
MUFA (g/day) #	25.8 ± 4.0	26.0 (19.0–31.9)	25.9 ± 4.2	25.7 (19.2–33.1)	0.738	27.4 ± 3.8	27.6 (21.1–32.8)	27.0 ± 4.2	27.6 (20.1–32.8)	0.200
PUFA (g/day) #	10.4 ± 1.5	10.2 (8.1–13.3)	10.2 ± 1.6	10.1 (7.8–12.8)	0.049	10.4 ± 1.8	10.2 (7.9–13.9)	10.3 ± 1.7	10.1 (7.8–13.1)	0.378
Fiber (g/day) #	15.5 ± 3.2	15.1 (11.3–20.0)	15.7 ± 3.0	15.4 (11.4–20.8)	0.423	16.5 ± 4.0	16.0 (11.1–22.1)	17.0 ± 3.7	16.6 (11.4–23.3)	0.148
Vitamin A (μg/day) #	844.7 ± 273.0	801.7 (482.6–1.273.6)	913.4 ± 278.0	876.1 (529.5–1.338.4)	<0.001	853.4 ± 307.2	805.9 (470.6–1.414.2)	893.8 ± 270.2	850.6 (514.5–1.386.6)	0.004
Vitamin D (μg/day) #	2.51 ± 1.12	2.31 (1.17–4.57)	2.89 ± 1.11	2.76 (1.36–4.90)	<0.001	2.42 ± 1.33	2.07 (0.94–4.72)	2.61 ± 1.20	2.43 (1.01–4.88)	0.001
Vitamin E (mg α–TE/day) #	8.7 ± 2.3	8.5 (5.6–12.8)	9.0 ± 2.3	8.8 (5.8–13.2)	0.238	8.9 ± 2.3	8.4 (6.0–12.7)	9.0 ± 2.1	8.8 (6.0–12.9)	0.332
Thiamin (mg/day) #	1.20 ± 0.18	1.18 (0.94–1.51)	1.27 ± 0.24	1.23 (0.96–1.73)	<0.001	1.18 ± 0.20	1.15 (0.90–1.56)	1.24 ± 0.21	1.21 (0.95–1.61)	0.001
Riboflavin (mg/day) #	1.71 ± 0.27	1.73 (1.24–2.16)	1.83 ± 0.33	1.79 (1.35–2.46)	<0.001	1.65 ± 0.31	1.63 (1.19–2.18)	1.77 ± 0.33	1.75 (1.32–2.38)	<0.001
Niacin (mg Eq. Niacin/day) #	29.1 ± 4.0	28.5 (23.3–36.0)	29.8 ± 4.3	29.5 (23.5–38.4)	0.033	28.7 ± 4.3	28.3 (22.4–36.9)	29.7 ± 4.2	29.3 (23.9–37.8)	0.002
Vitamin B_6_ (mg/day) #	1.81 ± 0.29	1.78 (1.35–2.30)	1.90 ± 0.34	1.87 (1.40–2.54)	0.004	1.76 ± 0.36	1.71 (1.24–2.43)	1.90 ± 0.38	1.88 (1.35–2.62)	<0.001
Vitamin B_12_ (μg/day) #	4.3 ± 1.0	4.2 (3.0–6.0)	4.3 ± 0.9	4.2 (3.1–5.9)	0.259	4.5 ± 1.4	4.2 (2.7–6.6)	4.5 ± 1.1	4.4 (2.8–6.3)	0.135
Folate (μg DFE b/day) #	222.7 ± 44.5	216.8 (160.7–305.3)	236.4 ± 48.7	229.3 (170.5–325.4)	0.001	230.1 ± 48.8	224.2 (160.0–317.6)	244.7 ± 51.4	242.2 (168.1–333.7)	0.003
Vitamin C (mg/day) #	98.1 ± 39.2	91.3 (46.9–167.9)	104.2 ± 37.8	98.3 (53.3–171.7)	0.047	96.5 ± 37.2	91.0 (47.2–163.5)	108.9 ± 40.4	103.4 (54.2–184.1)	0.001
Calcium (mg/day) #	935 ± 168	935 (652–1.208)	989 ± 171	975 (740–1.289)	<0.001	914 ± 152	924 (664–1.164)	955 ± 152	945 (711–1.214)	<0.001
Iron (mg/day) #	11.2 ± 1.8	10.9 (9.1–14.7)	11.6 ± 2.0	11.4 (8.9–15.6)	0.88	11.3 ± 1.9	11.0 (8.9–15.1)	11.6 ± 2.1	11.4 (8.8–15.9)	0.124
Potassium (mg/day)	2.568 ± 296	2.589 (2.054–3.056)	2.638 ± 343	2.616 (2.103–3.237)	0.001	2.494 ± 350	2.460 (1.954–3.080)	2.611 ± 333	2.590 (2.077–3.171)	<0.001
Sodium (mg/day) #	1.617 ± 319	1.572 (1.179–2.200)	1.650 ± 295	1.631 (1.246–2.181)	0.051	1.704 ± 284	1.696 (1.265–2.185)	1.722 ± 282	1.707 (1.301–2.204)	0.814
Zinc (mg/day) #	8.7 ± 1.0	8.6 (7.3–10.6)	8.8 ± 1.0	8.7 (7.2–10.5)	0.243	8.5 ± 1.1	8.3 (6.9–10.4)	8.6 ± 1.1	8.4 (7.1–10.8)	0.328
Magnesium (mg/day) #	236.1 ± 26.3	233.3 (194.2–282.3)	241.8 ± 30.9	238.8 (198.1–295.5)	0.021	238.7 ± 26.1	238.0 (196.7–282.1)	247.2 ± 26.4	245.6 (206.7–290.5)	<0.001
Selenium (μg/day) #	86.1 ± 15.4	85.5 (62.4–112.1)	87.2 ± 15.5	86.6 (63.8–114.6)	0.409	90.1 ± 15.8	88.8 (67.6–117.0)	91.8 ± 17.2	91.2 (66.3–118.6)	0.199
Iodine (μg/day) #	91.2 ± 15.0	89.7 (68.8–118.9)	92.2 ± 15.2	90.7 (68,7–118.1)	0.189	86.4 ± 14.2	85.2 (66.4–110.2)	90.0 ± 15.3	87.7 (67.7–117.0)	0.001
Nutrient Rich subscore #	768.9 ± 53.4	787.9 (668.2–823.3)	775.8 ± 51.7	796.6 (665,5–829,6)	0.001	768.7 ± 54.7	790.3 (665.2–827.4)	786,8 ± 44.2	807.1 (683.0–827.5)	<0.001
Limiting subscore #	284.0 ± 16.5	288.1 (253.1–300.0)	282.9 ± 17.7	287.3 (249.3–300.0)	0.773	284.1 ± 17.5	287.9 (247.4–300.0)	282.3 ± 19.0	285.7 (246.7–300.0)	0.255
NRF 9.3 score #	484.9 ± 52.8	499.1 (383.5–549.8)	492.9 ± 51.8	508.4 (388.0–559.9)	0.001	484.5 ± 56.4	499.2 (378.9–560.7)	504.4 ± 46.8	513.5 (407.1–568.2)	<0.001

Nutrients were adjusted using the residual method for total energy intake except for the percentage of energy from carbohydrates, proteins, and fats. † Significant differences (*p*–values) between WQB with GQB groups according to sex applying Student’s *t*–test or Mann–Whitney (#) U test. %E: Percentage of total energy, niacin was expressed as equivalents of niacin (preformed niacin + tryptophan/60). For vitamin A from β–carotene, a conversion factor of 1/6 was used, whereas for the other carotenoids, a conversion factor of 1/12 was used. Vitamin E was expressed as alpha––tocopherol equivalents (α–TE), and folate intake was calculated as µg of dietary folate equivalents (DFE) (food folate + 1.7 µg synthetic folic acid content of fortified food). NRF9.3 score was calculated based on daily intake of nine nutrients to encourage (nutrient rich subscore) and three nutrients to limit (limiting subscore). A higher NRF 9.3 score is indicative of a higher diet quality.

**Table 8:** Inadequate intakes of macronutrients and micronutrients in Spanish children *n* (%) by sex and type of breakfast (*n* = 1561).

	Boys							Girls						
	Worse Quality Breakfast (WQB)			Good Quality Breakfast (GQB)			*p* Value †	Worse Quality Breakfast (WQB)			Good Quality Breakfast (GQB)			*p* Value †
	<EAR, *n* (%)	<AMDR	>AMDR	<EAR, *n* (%)	<AMDR	>AMDR		<EAR, *n* (%)	<AMDR	>AMDR	<EAR, *n* (%)	<AMDR	>AMDR	
	[% > UL]			[% > UL]				[% > UL]			[% > UL]			
Carbohydrates, total (g/day)	0 (0.0)			0 (0.0)			–	0 (0.0)			0 (0.0)			–
Protein (g/day)	0 (0.0)			0 (0.0)			–	0 (0.0)			0 (0.0)			–
Carbohydrates, total (%E)		395 (100)	0 (0)		429 (100)	0 (0)	–		385 (99.7)	0 (0)		351 (100.0)	0 (0)	0.340
Protein (%E)		0 (0)	3 (0.8)		0 (0)	8 (1.9)	0.167		0 (0)	4 (1.0)		0 (0)	18 (5.1)	0.001
Fat, total (%E)		0 (0.0)	159 (40.3)		0 (0)	128 (20.8)	0.002		0 (0.0)	160 (41.5)		0 (0.0)	102 (29.1)	<0.001
Vitamin A (μg/day)	11 (2.8)			9 (2.1)			0.522	9 (2.3)			8 (2.3)			0.962
Vitamin D (μg/day)	395 (100) [0.0]			429 (100) [0.0]			–	384 (99.5) [0.0]			351 (100) [0.0]			0.177
Vitamin E (mg α–TE/day)	122 (30.9) [0.0]			127 (29.6) [0.0]			0.689	155 (40.2) [0.0]			99 (28.2) [0.0]			0.001
Thiamin (mg/day)	0 (0)			2 (0.5)			0.174	4 (1)			2 (0.6)	0.175	0.47	0.482
Riboflavin (mg/day)	0 (0)			0 (0)			–	2 (0.5)			0 (0)		0.176	0.177
Niacin (mg Eq. Niacin/day)	0 (0)			0 (0)			–	0 (0)			0 (0)			–
Vitamin B_6_ (mg/day)	0 (0) [0.0]			0 (0) [0.0]			–	0 (0) [0.0]			1 (0.3) [0.0]			0.294
Vitamin B_12_ (μg/day)	0 (0)			0 (0)			–	1 (0.3)			0 (0)			0.340
Folate (μg DFE/day)	136 (34.4) [1.3]			109 (25.4) [1.4]			0.005	213 (55.2) [0.0]			204 (58.1) [0.0]			<0.001
Vitamin C (mg/day)	6 (1.5) [0.0]			5 (1.2) [0.0]			0.659	4 (1) [0.0]			2 (0.6) [0.0]			0.482
Calcium (mg/day)	157 (39.7) [0.0]			119 (27.7) [0.0]			<0.001	204 (52.8) [0.0]			130 (37.0) [0.0]			<0.001
Iron (mg/day)	0 (0) [0.0]			0 (0) [0.0]			–	0 (0) [0.0]			2 (0.6) [0.0]			0.138
Potassium (mg/day) ‡	393 (99.5)			421 (98.1)			0.075	384 (99.5)			347 (98.9)			0.348
Sodium (mg/day) ‡	45 (11.4)			46 (10.7)			0.759	64 (16.6)			55 (15.7)			0.737
Zinc (mg/day)	2 (0.5) [12.2]			2 (0.5) [23.8]			0.934	19 (4.9) [9.6]			6 (1.7) [18.8]			0.016
Magnesium (mg/day)	40 (10.1)			63 (14.7)			0.048	68 (17.6)			35 (10)			0.003
Selenium (μg/day)	0 (0) [0.3]			0 (0) [1.6]				0 (0) [1.0]			0 (0) [2.3]			–
Iodine (μg/day)	41 (10.4) [0.0]			51 (11.9) [0.0]			0.492	96 (24.9) [0.0]			58 (16.5) [0.0]			0.005

† The chi square test has been applied. EAR: Estimated average requirement, AMDR: Acceptable Macronutrient Distribution Range. UL: Upper limit, ‡ Adequate intake data are considered since EAR data are not available. *p*-value UL = all differences are not significant except in the case of zinc, *p* < 0.001 for both boys and girls, and in the case of selenium in boys *p* = 0.044.

## Supplementary Materials

The following are available online at https://www.mdpi.com/2072-6643/12/12/3772/s1, Table S1: Energy and nutrients provided at breakfast, Table S2: Contribution of breakfast intake to coverage of recommended nutrient intakes (%), Table S3: Energy and nutrients provided at breakfast. Results for plausible reporters (*n* = 1311), Table S4: Contribution of breakfast intake to coverage of recommended nutrient intakes (%). Results for plausible reporters (*n* = 1311), Table S5: Breakfast food consumption by age group (g/day). Results for plausible reporters (*n* = 1311), Table S6: Breakfast Quality Index (BQI) components and number of children meeting the criteria (n, %) by age group. Results for plausible reporters (*n* = 1311), Table S7: Breakfast Quality Index (BQI) components and number of children meeting the criteria (*n*, %). Results for plausible reporters (*n* = 1311), Table S8: Usual intakes (from food and beverage sources only) adjusted by energy of macronutrients and micronutrients in Spanish children and adolescents by sex and type of breakfast. Results for plausible reporters (*n* = 1311), Table S9: Inadequate intakes of macronutrients and micronutrients in Spanish children (*n*, %) by sex and type of breakfast. Results for plausible reporters (*n* = 1311).
