# Supplementary material for: Breakfast Habits of a Representative Sample of the Spanish Child and Adolescent Population (The ENALIA Study): Association with Diet Quality
Source: Nutrients. 2020 Dec 8;12(12):3772. doi: 10.3390/nu12123772 (PMC7763817; doi:10.3390/nu12123772)
Supplement: Supplementary file 1 [file nutrients-12-03772-s001.pdf]

Table S1. Energy and nutrients provided at breakfast by type of breakfast.

Intake/day	Worse quality breakfast (BQI< P66)		Good quality breakfast (BQI ≥ P66)		
	X ± SD	P50 (P5–P95)	X ± SD	P50 (P5–P95)	p-Value
Energy (kcal)	325 ± 146	303 (134 - 591)	338 ± 134	313 (170 - 599)	0.208
Protein (g)	10.3 ± 4.0	10.0 (4.6 - 17.3)	11.7 ± 4.7	10.9 (6.0 - 20.0)	<0.001
Protein (%E)	13.6 ± 4.3	12.8 (7.8 - 22.1)	14.4 ± 3.7	13.7 (9.3 - 21.3)	<0.001
Carbohydrates (g)	43.0 ± 20.4	39.9 (15.7 - 77.1)	45.1 ± 21.2	41.2 (19.8 - 86.2)	0.633
Carbohydrates (%E)	52.9 ± 9.3	52.4 (38.9 - 68.8)	53.0 ± 10.1	53.2 (37.4 - 70.4)	0.919
Total sugars (g)	26.4 ± 11.3	24.1 (11.6 - 48.6)	24.5 ± 11.7	21.0 (11.9 - 48.0)	<0.001
Total sugars (%E)	34.3 ± 9.7	33.6 (20.2 - 51.0)	29.6 ± 8.6	29.2 (16.0 - 44.5)	<0.001
Fat, total (g)	12.0 ± 7.2	10.6 (3.2 - 25.6)	11.8 ± 6.2	10.8 (4.0 - 23.0)	0.704
Fat, total (%E)	32.4 ± 9.9	33.0 (14.7 - 46.7)	31.5 ± 10.5	31.7 (13.6 - 47.2)	0.215
SFA (g)	5.89 ± 3.73	5.42 (0.25 - 13.11)	5.55 ± 3.05	5.38 (0.84 - 11.04)	0.374
MUFA (g)	3.51 ± 2.63	2.96 (0.11 - 8.59)	3.86 ± 2.70	3.37 (0.47 - 8.46)	0.007
PUFA (g)	1.24 ± 1.42	0.79 (0.10 - 4.20)	1.13 ± 1.20	0.80 (0.16 - 3.26)	0.365
Fiber (g)	1.99 ± 2.81	1.32 (0.19 - 5.78)	2.13 ± 2.14	1.51 (0.35 - 6.04)	0.025
Vitamin A (μg)	146 ± 120	116 (27 - 379)	180 ± 132	158 (38 - 408)	<0.001
Vitamin D (μg)	0.57 ± 0.81	0.16 (0.00 - 2.18)	0.86 ± 1.00	0.50 (0.03 - 2.91)	<0.001
Vitamin E (mg α–TE)	1.10 ± 1.28	0.54 (0.11 - 3.79)	1.27 ± 1.19	0.81 (0.20 - 3.73)	<0.001
Thiamin (mg)	0.23 ± 0.18	0.17 (0.07 - 0.65)	0.31 ± 0.24	0.23 (0.09 - 0.73)	<0.001
Riboflavin (mg)	0.53 ± 0.27	0.50 (0.12 - 1.05)	0.62 ± 0.31	0.55 (0.23 - 1.16)	<0.001
Niacin (mg Eq. Niacin)	3.47 ± 2.08	3.00 (1.17 - 7.38)	4.64 ± 2.97	3.91 (1.77 - 9.64)	<0.001
Vitamin B6 (mg)	0.26 ± 0.25	0.17 (0.07 - 0.84)	0.37 ± 0.34	0.24 (0.10 - 0.99)	<0.001
Vitamin B12 (μg)	0.77 ± 0.35	0.75 (0.13 - 1.40)	0.84 ± 0.36	0.81 (0.30 - 1.45)	0.003
Folate (μg DFE)	37.7 ± 30.7	27.2 (7.4 - 96.7)	52.3 ± 40.6	41.3 (12.5 - 127.1)	<0.001
Vitamin C (mg)	14.5 ± 21.8	4.3 (1.0 - 63.9)	22.8 ± 30.5	11.2 (1.2 - 92.0)	<0.001
Calcium (mg)	311 ± 122	309 (95 - 513)	340 ± 118	326 (170 - 545)	0.001
Iron (mg)	1.85 ± 1.70	1.36 (0.28 - 5.23)	2.20 ± 1.78	1.71 (0.51 - 5.49)	<0.001
Potassium (mg)	439 ± 178	428 (167 - 757)	489 ± 205	447 (227 - 880)	0.002
Sodium (mg)	239 ± 140	212 (80 - 486)	309 ± 196	266 (114 - 663)	<0.001
Zinc (mg)	1.53 ± 1.14	1.35 (0.45 - 2.79)	1.57 ± 0.81	1.43 (0.73 - 2.70)	0.039
Magnesium (mg)	43.1 ± 21.9	39.8 (15.0 - 83.1)	47.6 ± 22.6	42.0 (19.4 - 91.8)	0.009
Selenium (μg)	8.08 ± 6.73	6.32 (0.65 - 21.22)	10.93 ± 7.73	9.15 (2.16 - 25.97)	<0.001
Iodine (μg)	21.3 ± 9.4	21.5 (5.3 - 38.1)	22.6 ± 8.0	22.3 (9.0 - 36.2)	0.019

Significant differences between worse and good quality breakfast groups according to sex, applying Mann Whitney test. Variables does not follow a normal distribution. SD: standard deviation. Niacin was expressed as equivalents of niacin (preformed niacin + tryptophan/60). For vitamin A from β–carotene, a conversion factor of 1/6 was used, whereas for the other carotenoids, a conversion factor of 1/12 was used. Vitamin E was expressed as alpha–tocopherol equivalents (α–TE), and folate intake was calculated as μg of dietary folate equivalents (DFE) (food folate + 1.7 μg synthetic folic acid content of fortified food).

Table S2. Contribution of breakfast intake to coverage of recommended nutrient intakes (%).

	Total		Worse quality breakfast (BQI< P66)		Good quality breakfast (BQI ≥ P66)		
	X ± SD	P50 (P5–P95)	X ± SD	P50 (P5–P95)	X ± SD	P50 (P5–P95)	p- Value
Protein	46.0 ± 23.6	41.5 (15.7 - 91.2)	41.0 ± 20.1	38.0 (14.3 - 77.9)	51.4 ± 25.9	46.9 (19.2 - 100.0)	<0.001
Carbohydrates	44.0 ± 20.8	40.9 (17.4 - 81.2)	43.0 ± 20.4	39.9 (15.7 - 77.1)	45.1 ± 21.2	41.2 (19.8 - 86.2)	0.633
Calcium	37.8 ± 15.9	36.1 (14.0 - 65.6)	34.5 ± 14.6	33.3 (10.2 - 59.3)	41.4 ± 16.5	38.3 (19.3 - 72.7)	<0.001
Iron	43.2 ± 41.8	29.6 (6.7 - 139.8)	39.6 ± 41.6	26.1 (4.6 - 138.6)	47.2 ± 41.7	33.0 (10.7 - 141.6)	<0.001
Potassium †	11.4 ± 4.3	11.0 (4.9 - 19.1)	10.6 ± 4.1	10.2 (4.1 - 18.0)	12.2 ± 4.4	11.5 (6.5 - 20.0)	<0.001
Sodium †	20.4 ± 11.8	18.0 (7.1 - 41.5)	17.7 ± 9.6	16.0 (6.3 - 34.8)	23.4 ± 13.1	20.5 (9.8 - 47.4)	<0.001
Zinc	32.9 ± 25.6	26.0 (9.1 - 88.9)	31.5 ± 27.6	23.7 (7.2 - 93.7)	34.5 ± 23.1	28.3 (12.8 - 81.3)	<0.001
Magnesium	31.3 ± 16.5	28.7 (10.0 - 61.8)	28.8 ± 15.9	26.0 (8.4 - 58.6)	34.0 ± 16.7	31.9 (11.6 - 65.7)	<0.001
Selenium	31.6 ± 22.6	26.3 (5.1 - 74.5)	26.6 ± 21.3	21.1 (3.0 - 65.9)	37.0 ± 22.8	31.2 (9.5 - 78.9)	<0.001
Iodine	30.3 ± 12.1	30.6 (8.4 - 50.1)	29.6 ± 13.1	29.7 (6.6 - 51.3)	31.1 ± 10.9	31.2 (11.3 - 48.0)	0.028
Vitamin A	45.8 ± 36.4	37.7 (7.8 - 107.6)	41.4 ± 36.2	32.7 (6.4 - 100.9)	50.6 ± 36.0	44.0 (9.2 - 109.3)	<0.001
Vitamin D	7.1 ± 9.2	2.7 (0.2 - 25.4)	5.7 ± 8.1	1.6 (0.0 - 21.8)	8.6 ± 10.0	5.0 (0.3 - 29.1)	<0.001
Vitamin E	17.0 ± 20.4	8.6 (1.6 - 63.8)	15.7 ± 20.7	6.7 (1.2 - 63.2)	18.4 ± 20.0	10.7 (2.4 - 63.8)	<0.001
Thiamin	44.1 ± 35.6	31.8 (11.4 - 116.7)	37.9 ± 32.1	27.3 (10.0 - 107.3)	51.0 ± 37.9	39.1 (13.0 - 123.2)	<0.001
Riboflavin	91.6 ± 49.9	86.1 (23.3 - 184.5)	83.7 ± 46.6	78.3 (15.5 - 170.5)	100.4 ± 52.0	92.7 (31.5 - 192.8)	<0.001
Vitamin B6	49.2 ± 46.7	31.9 (10.7 - 142.8)	41.3 ± 41.8	26.3 (9.5 - 132.6)	57.9 ± 50.3	38.9 (12.5 - 156.0)	<0.001
Niacin	53.9 ± 34.2	46.1 (16.8 - 118.6)	46.3 ± 29.7	39.9 (13.8 - 105.3)	62.3 ± 36.8	53.4 (22.0 - 136.8)	<0.001
Vitamin B12	66.8 ± 32.5	64.8 (17.9 - 125.0)	62.0 ± 30.4	60.0 (11.3 - 115.8)	72.2 ± 33.9	69.3 (22.5 - 135.0)	<0.001
Folate	21.2 ± 18.3	15.3 (3.7 - 57.1)	18.2 ± 16.4	12.7 (3.0 - 51.7)	24.4 ± 19.6	18.5 (4.6 - 63.2)	<0.001
Vitamin C	66.0 ± 93.2	24.4 (2.8 - 260.4)	53.7 ± 85.6	14.8 (2.3 - 244.3)	79.5 ± 99.4	40.8 (3.9 - 286.5)	<0.001

Significant differences between good– and low–quality breakfast groups according to sex, applying Mann Whitney test. Variables does not follow a normal distribution. The recommended nutrient intakes were dietary references intakes from IoM. † DRI used is an adequate intake. SD: standard deviation.

Table S3. Energy and nutrients provided at breakfast. Results for plausible reporters (n=1311).

Intake/day	Worse quality breakfast (BQI< P66)		Good quality breakfast (BQI ≥ P66)		
	X ± SD	P50 (P5–P95)	X ± SD	P50 (P5–P95)	p-Value
Energy (kcal)	328 ± 144	300 (144 - 591)	339 ± 134	316 (170 - 595)	0.400
Protein (g)	10.4 ± 4.0	10.0 (5.0 - 17.7)	11.7 ± 4.9	10.8 (5.9 - 20.0)	0.001
Protein (%E)	13.5 ± 4.0	12.8 (8.1 - 21.6)	14.2 ± 3.6	13.6 (9.3 - 20.8)	<0.001
Carbohydrates (g)	43.3 ± 20.4	39.9 (16.9 - 78.9)	44.7 ± 20.7	40.9 (19.7 - 87.0)	0.842
Carbohydrates (%E)	52.8 ± 9.2	52.3 (38.5 - 68.7)	52.3 ± 9.7	52.7 (37.5 - 69.5)	0.255
Total sugars (g)	26.7 ± 11.3	24.3 (12.2 - 48.8)	24.4 ± 11.6	20.9 (11.9 - 47.0)	<0.001
Total sugars (%E)	34.1 ± 9.3	33.6 (20.2 - 49.5)	29.3 ± 8.3	28.9 (15.9 - 44.1)	<0.001
Fat, total (g)	12.1 ± 7.0	10.7 (3.5 - 25.5)	12.1 ± 6.2	11.1 (4.3 - 23.1)	0.435
Fat, total (%E)	32.6 ± 9.7	33.0 (16.2 - 46.8)	32.4 ± 9.9	32.5 (15.5 - 47.2)	0.945
SFA (g)	5.95 ± 3.65	5.48 (0.18 - 13.13)	5.71 ± 2.98	5.53 (1.22 - 11.01)	0.653
MUFA (g)	3.50 ± 2.57	3.03 (0.10 - 8.38)	3.96 ± 2.71	3.55 (0.71 - 8.46)	0.002
PUFA (g)	1.21 ± 1.29	0.79 (0.11 - 3.75)	1.15 ± 1.21	0.82 (0.18 - 3.26)	0.459
Fiber (g)	2.02 ± 2.95	1.32 (0.26 - 5.58)	2.06 ± 2.11	1.43 (0.35 - 5.32)	0.271
Vitamin A (μg)	151 ± 120	126 (32 - 382)	182 ± 133	159 (39 - 420)	<0.001
Vitamin D (μg)	0.61 ± 0.83	0.20 (0.02 - 2.25)	0.85 ± 0.95	0.50 (0.04 - 2.87)	<0.001
Vitamin E (mg α–TE)	1.13 ± 1.32	0.54 (0.11 - 3.93)	1.27 ± 1.19	0.83 (0.20 - 3.73)	<0.001
Thiamin (mg)	0.23 ± 0.19	0.17 (0.08 - 0.65)	0.30 ± 0.23	0.23 (0.09 - 0.71)	<0.001
Riboflavin (mg)	0.54 ± 0.26	0.50 (0.16 - 1.07)	0.62 ± 0.30	0.55 (0.23 - 1.13)	<0.001
Niacin (mg Eq. Niacin)	3.56 ± 2.10	3.02 (1.35 - 7.81)	4.60 ± 2.86	3.86 (1.76 - 9.46)	<0.001
Vitamin B6 (mg)	0.27 ± 0.26	0.17 (0.08 - 0.86)	0.36 ± 0.32	0.24 (0.09 - 0.98)	<0.001
Vitamin B12 (μg)	0.78 ± 0.34	0.75 (0.26 - 1.36)	0.84 ± 0.36	0.81 (0.30 - 1.45)	0.021
Folate (μg DFE)	39.1 ± 31.3	28.4 (8.0 - 99.9)	51.4 ± 39.2	41.1 (12.3 - 126.4)	<0.001
Vitamin C (mg)	14.5 ± 21.6	4.4 (1.0 - 64.3)	21.9 ± 29.5	10.9 (1.2 - 89.8)	<0.001
Calcium (mg)	317 ± 119	312 (124 - 519)	340 ± 116	326 (168 - 545)	0.014
Iron (mg)	1.92 ± 1.74	1.44 (0.29 - 5.32)	2.17 ± 1.73	1.68 (0.49 - 5.43)	0.007
Potassium (mg)	440 ± 176	428 (171 - 757)	485 ± 205	444 (223 - 861)	0.015
Sodium (mg)	239 ± 137	213 (82 - 473)	305 ± 191	262 (114 - 660)	<0.001
Zinc (mg)	1.56 ± 1.16	1.35 (0.51 - 2.84)	1.56 ± 0.80	1.41 (0.71 - 2.71)	0.370
Magnesium (mg)	43.3 ± 22.1	39.7 (15.4 - 83.5)	46.5 ± 22.2	41.2 (19.2 - 86.8)	0.116
Selenium (μg)	8.07 ± 6.60	6.32 (0.52 - 20.48)	10.85 ± 7.94	9.05 (2.10 - 26.53)	<0.001
Iodine (μg)	21.7 ± 9.1	21.7 (6.7 - 37.9)	22.6 ± 8.0	22.3 (8.3 - 36.4)	0.142

Significant differences between worse and good quality breakfast groups according to sex, applying Mann Whitney test. Variables does not follow a normal distribution. Niacin was expressed as equivalents of niacin (preformed niacin + tryptophan/60). For vitamin A from β–carotene, a conversion factor of 1/6 was used, whereas for the other carotenoids, a conversion factor of 1/12 was used. Vitamin E was expressed as alpha–tocopherol equivalents (α–TE), and folate intake was calculated as μg of dietary folate equivalents (DFE) (food folate + 1.7 μg synthetic folic acid content of fortified food). SD: standard deviation.

Table S4. Contribution of breakfast intake to coverage of recommended nutrient intakes (%).Results for plausible reporters (n=1311).

	**Worse quality breakfast (BQI< P66)**		**Good quality breakfast (BQI ≥ P66)**		
	**X ± SD**	**P50 (P5–P95)**	**X ± SD**	**P50 (P5–P95)**	**p-Value**
Protein	42.4 ± 20.0	39.5(16.2 - 79.8)	53.3 ± 25.4	49.6(20.8 - 100.3)	<0.001
Carbohydrates	43.3 ± 20.4	39.9(16.9 - 78.9)	44.7 ± 20.7	40.9(19.7 - 87.0)	0.842
Calcium	35.6 ± 14.4	34.8(13.8 - 60.4)	42.2 ± 16.0	39.0(23.8 - 71.2)	<0.001
Iron	41.9 ± 42.8	27.1(5.6 - 143.8)	47.9 ± 41.2	33.6(10.7 - 143.3)	<0.001
Potassium †	10.7 ± 4.0	10.4(4.5 - 18.1)	12.2 ± 4.3	11.5(6.5 - 19.6)	<0.001
Sodium †	17.8 ± 9.4	16.1(6.6 - 34.5)	23.4 ± 12.9	20.5(10.0 - 47.8)	<0.001
Zinc	32.8 ± 28.7	24.4(8.5 - 95.0)	35.5 ± 23.3	29.5(13.3 - 82.6)	<0.001
Magnesium	29.7 ± 15.9	26.8(9.5 - 58.7)	35.1 ± 16.6	33.3(12.3 - 66.7)	<0.001
Selenium	27.0 ± 21.1	22.0(3.0 - 65.2)	37.8 ± 23.3	31.7(9.5 - 79.3)	<0.001
Iodine	30.3 ± 12.6	30.2(8.6 - 51.0)	31.6 ± 10.9	31.8(11.0 - 49.3)	0.098
Vitamin A	43.3 ± 36.4	33.5(7.8 - 100.9)	52.5 ± 36.2	45.9(10.5 - 109.3)	<0.001
Vitamin D	6.1 ± 8.3	2.0(0.2 - 22.5)	8.5 ± 9.5	5.0(0.4 - 28.7)	<0.001
Vitamin E	16.4 ± 21.5	6.9(1.4 - 66.2)	19.0 ± 20.3	11.1(2.6 - 64.8)	<0.001
Thiamin	39.7 ± 32.6	28.1(11.1 - 111.9)	51.6 ± 36.4	39.6(13.5 - 123.2)	<0.001
Riboflavin	87.1 ± 45.9	82.8(25.7 - 176.7)	103.2 ± 49.9	96.1(36.2 - 192.5)	<0.001
Vitamin B6	43.5 ± 42.4	27.3(10.7 - 138.5)	58.5 ± 48.9	39.3(13.1 - 156.8)	<0.001
Niacin	48.1 ± 29.5	41.3(16.4 - 111.2)	63.4 ± 35.4	54.3(23.3 - 136.8)	<0.001
Vitamin B12	63.9 ± 29.2	63.5(19.6 - 115.8)	74.5 ± 33.3	72.9(24.8 - 135.0)	<0.001
Folate	19.1 ± 16.8	13.4(3.3 - 54.4)	24.8 ± 19.5	18.9(4.6 - 64.3)	<0.001
Vitamin C	55.9 ± 87.1	15.4(2.6 - 244.3)	79.8 ± 98.9	41.1(3.6 - 293.3)	<0.001

Significant differences between good– and low–quality breakfast groups according to sex, applying Mann Whitney test. Variables do not follow a normal distribution. The recommended nutrient intakes were dietary references intakes from IoM. † DRI used is an adequate intake. SD: standard deviation.

Table S5. Breakfast food consumption by age group (g/day). Results for plausible reporters (n=1311).

		1–3 years	4–8 years	9–13 years	14–17 years	p–Value †	14–17 years		p–Value $
							Boys	Girls	
N		262	492	479	232		120	111	
Dairy	X ± SD	141.7 ± 120.5 ^{a}	202.6 ± 73.3 ^{b}	199.0 ± 77.8 ^{b}	196.0 ± 92.3 ^{b}	<0.001	216.7 ± 87.5	173.6 ± 92.6	0.003
	P50 (P5–P95)	141.2 (0.0 - 297.9)	216.5 (38.0 - 300.0)	207.0 (47.0 - 313.9)	204.8 (0.0 - 345.8)		218.9 (65.0 - 350.4)	199.5 (0.0 - 292.9)	
Cereals	X ± SD	15.1 ± 30.3 ^{a}	18.7 ± 27.2 ^{a,b}	22.7 ± 28.7 ^{b}	21.1 ± 37.3 ^{a,b}	<0.001	20.4 ± 26.9	21.9 ± 46.1	0.608
	P50 (P5–P95)	5.5 (0.0 - 51.0)	12.3 (0.0 - 56.3)	16.9 (0.0 - 70.7)	10.2 (0.0 - 71.6)		12.4 (0.0 - 71.1)	9.6 (0.0 - 71.6)	
Fruits	X ± SD	7.0 ± 38.2 ^{a,b}	7.9 ± 35.2 ^{a}	19.1 ± 76.9 ^{b,c}	24.4 ± 83.1 ^{c}	<0.001	24.5 ± 89.1	24.3 ± 76.4	0.699
	P50 (P5–P95)	0.0 (0.0 - 45.5)	0.0 (0.0 - 66.4)	0.0 (0.0 - 126.0)	0.0 (0.0 - 126.2)		0.0 (0.0 - 118.1)	0.0 (0.0 - 126.2)	
Protein foods	X ± SD	0.8 ± 4.0 ^{a}	1.4 ± 5.2 ^{a}	1.8 ± 6.4 ^{a}	3.6 ± 16.2 ^{b}	0.018	4.9 ± 21.4	2.2 ± 7.2	0.747
	P50 (P5–P95)	0.0 (0.0 - 5.3)	0.0 (0.0 - 12.2)	0.0 (0.0 - 13.4)	0.0 (0.0 - 23.0)		0.0 (0.0 - 30.0)	0.0 (0.0 - 18.8)	
Added Products	X ± SD	3.2 ± 5.6 ^{a}	7.2 ± 8.6 ^{b}	7.7 ± 10.3 ^{b}	7.4 ± 6.9 ^{b}	<0.001	7.4 ± 6.6	7.4 ± 7.2	0.794
	P50 (P5–P95)	0.0 (0.0 - 13.8)	5.6 (0.0 - 20.3)	5.4 (0.0 - 19.2)	6.3 (0.0 - 21.7)		6.5 (0.0 - 21.7)	5.6 (0.0 - 24.1)	
Fats and oils	X ± SD	0.6 ± 2.0 ^{a}	1.9 ± 3.7 ^{b}	2.1 ± 4.1 ^{b}	1.6 ± 3.4 ^{b}	<0.001	1.9 ± 3.8	1.2 ± 2.7	0.326
	P50 (P5–P95)	0.0 (0.0 - 4.4)	0.0 (0.0 - 10.6)	0.0 (0.0 - 11.4)	0.0 (0.0 - 8.8)		0.0 (0.0 - 9.2)	0.0 (0.0 - 7.3)	
Baby foods	X ± SD	68.1 ± 106.2 ^{a}	4.4 ± 28.4 ^{b}	0.8 ± 11.3 ^{b}	0.8 ± 8.3 ^{b}	<0.001	0.0 ± 0.0	1.6 ± 12.0	0.124
	P50 (P5–P95)	8.6 (0.0 - 287.5)	0.0 (0.0 - 4.4)	0.0 (0.0 - 0.0)	0.0 (0.0 - 0.0)		0.0 (0.0 - 0.0)	0.0 (0.0 - 0.0)	
Bakery	X ± SD	14.3 ± 29.4 ^{a}	22.0 ± 34.5 ^{b}	20.9 ± 28.4 ^{b}	21.3 ± 29.2 ^{a,b}	<0.001	18.0 ± 22.8	24.9 ± 34.6	0.297
	P50 (P5–P95)	0.0 (0.0 - 63.8)	9.8 (0.0 - 93.9)	11.2 (0.0 - 75.6)	10.5 (0.0 - 74.9)		8.3 (0.0 - 58.4)	11.8 (0.0 - 98.3)	
Juices	X ± SD	8.8 ± 33.1 ^{a}	14.9 ± 42.8 ^{a}	23.1 ± 53.2 ^{b}	26.3 ± 52.4 ^{b}	<0.001	24.8 ± 51.9	27.9 ± 53.0	0.434
	P50 (P5–P95)	0.0 (0.0 - 99.3)	0.0 (0.0 - 104.3)	0.0 (0.0 - 140.8)	0.0 (0.0 - 154.2)		0.0 (0.0 - 159.7)	0.0 (0.0 - 151.9)	
Other foods	X ± SD	0.6 ± 2.9 ^{a}	0.6 ± 2.9 ^{a}	0.8 ± 3.3 ^{a}	1.3 ± 5.1 ^{a}	0.001	1.8 ± 6.3	0.7 ± 3.4	0.554
	P50 (P5–P95)	0.0 (0.0 - 4.6)	0.0 (0.0 - 4.3)	0.0 (0.0 - 8.3)	0.0 (0.0 - 8.2)		0.0 (0.0 - 10.6)	0.0 (0.0 - 1.3)	

† Significant differences between age groups are shown, applying Kruskal Wallis test. $ Significant differences between sex groups in adolescents (14–17 years old) are shown, applying Mann–Whitney test. Variables do not follow a normal distribution. SD: standard deviation. Different superscripts (a, b, c) denotes significant difference between groups ($p < 0.05$). Added products: Other foods that can help make this food more appetizing (sugar, honey, powdered chocolate, jam...).

Table S6. Breakfast Quality Index (BQI) components and number of children meeting the criteria (n,%) by age group. Results for plausible reporters (n=1311).

	Total sample	1–3 years	4–8 years	9–13 years	14–17 years	p–Value †	14–17 years		p–Value †
							Boys	Girls	
N	1465	262	492	479	232		120	111	
BQI Item, n (%)									
Cereals and derivate consumption	816 (62.2)	137 (52.4) [a]	314 (63.9) [b,c]	339 (70.7) [b]	138 (59.4) [a,c]	< 0.001	69 (57.4)	69 (61.6)	0.524
Fruits and/or vegetables consumption	181 (13.8)	22 (8.5) [a]	56 (11.4) [a]	87 (18.1) [b]	48 (20.9) [b]	< 0.001	25 (20.8)	23 (20.9)	0.983
Dairy products consumption	1137 (86.7)	175 (66.7) [a]	472 (96.0) [b]	461 (96.1) [b]	214 (92.4) [b]	< 0.001	116 (96.0)	98 (88.4)	0.031
Food rich in simple sugars (< 5 %E)	557 (42.5)	159 (60.8) [a]	219 (44.6) [b]	151 (31.5) [c]	58 (24.9) [c]	< 0.001	23 (18.8)	35 (31.4)	0.028
Include MUFA–rich fats	169 (12.9)	20 (7.7) [a]	76 (15.4) [b]	75 (15.7) [b]	31 (13.3) [a,b]	0.012	18 (14.9)	13 (11.6)	0.479
MUFA/SFA ratio (≥2:1)	14 (1.1)	3 (1.1) [a]	4 (0.8) [a]	2 (0.5) [a]	5 (2.1) [a]	0.158	2 (2.0)	3 (2.3)	0.596
Energy intake from breakfast (20–25 %E)	208 (15.9)	33 (12.7) [a]	65 (13.3) [a]	90 (18.9) [a]	48 (20.5) [a]	< 0.001	19 (15.8)	29 (25.6)	< 0.001
Fruits. cereals and dairy product in the breakfast	100 (7.6)	9 (3.4) [a]	33 (6.6) [a,b]	50 (10.5) [b,c]	30 (12.8) [c]	< 0.001	17 (13.9)	13 (11.6)	0.596
Calcium (>20 % RDA)	1138 (86.8)	239 (91.1) [a]	449 (91.2) [a]	387 (80.7) [b]	193 (83.0) [b]	< 0.001	111 (92.1)	82 (73.3)	<0.001
Absence of butter or margarine	1273 (97.1)	261 (99.8) [a]	476 (96.8) [b]	459 (95.8) [b]	221 (95.2) [b]	0.021	113 (94.1)	108 (96.5)	0.418
Breakfast quality BQI score (0–10), X ± SD	4.27 ± 1.24	3.96 ± 1.22 [a]	4.40 ± 1.20 [b]	4.47 ± 1.24 [b]	4.30 ± 1.25 [b]	< 0.001	4.28 ± 1.27	4.33 ± 1.23	0.466

†The chi square test has been applied. Different superscripts (a,b,c) denotes significant differences between groups of age in the two-sided equality test for column proportions.
RDA: Recommended Dietary Allowance (IOM, 2000).

Table S7. Breakfast Quality Index (BQI) components and number of children meeting the criteria (n,%). Results for plausible reporters (n=1311).

	**WQB (BQI < P66)**	**GQB (BQI ≥P66)**	
	**(n=656)**	**(n=655)**	**p-Value**
BQI Item. n (%)			
Cereals and derivate consumption	328 (42.5)	601 (86.5)	<0.001
Fruits and/or vegetables consumption	41 (5.3)	173 (24.9)	<0.001
Dairy products consumption	667 (86.5)	655 (94.3)	<0.001
Food rich in simple sugars (< 5 %E)	216 (28)	372 (53.6)	<0.001
Include MUFA–rich fats	10 (1.3)	192 (27.6)	<0.001
MUFA/SFA ratio (≥2:1)	0 (0)	14 (2.1)	<0.001
Energy intake from breakfast (20–25 %E)	55 (7.1)	182 (26.2)	<0.001
Fruits, cereals and dairy product in the breakfast	0 (0)	122 (17.5)	<0.001
Calcium (>20 % RDA)	610 (79.1)	657 (94.6)	<0.001
Absence of butter or margarine	730 (94.8)	687 (99)	<0.001
Breakfast quality BQI score (0–10)	3.51 ± 0.68	5.24 ± 1.07	<0.001

The chi square test has been applied. RDA: Recommended Dietary Allowance (IOM, 2000). WQB: Worse quality breakfast, GQB: Good quality breakfast.

Table S8. Usual intakes (from food and beverage sources only) adjusted by energy of macronutrients and micronutrients in Spanish children and adolescents by sex and type of breakfast. Results for plausible reporters (n=1311).

	**Boys**					**Girls**				
	**WORSE QUALITY BREAKFAST**		**GOOD–QUALITY BREAKFAST**			**WORSE QUALITY BREAKFAST**		**GOOD–QUALITY BREAKFAST**		
	**Mean ± SD**	**Median (P5–P95)**	**Mean ± SD**	**Median (P5–P95)**	**p value**	**Mean ± SD**	**Median (P5–P95)**	**Mean ± SD**	**Median (P5–P95)**	p value
Energy (kcal)#	2.023 ± 356	2.036 (1.452 - 2.622)	2.022 ± 480	1.986 (1.325 - 2.881)	0.034	1.807 ± 263	1.803 (1.366 - 2.241)	1.738 ± 306	1.728 (1.249 - 2.267)	<0.001
Protein g #	75.5 ± 7.5	74.5 (64.6 - 87.7)	76.3 ± 7.6	76.2 (63.6 - 90.0)	0.099	75.2 ± 7.9	74.8 (62.3 - 89.7)	76.6 ± 7.8	75.6 (65.2 - 92.1)	0.023
Protein % #	16.6 ± 1.7	16.5 (14.3 - 19.3)	16.8 ± 1.7	16.8 (14.2 - 19.7)	0.065	16.6 ± 1.8	16.6 (13.7 - 19.9)	17.0 ± 1.8	16.8 (14.6 - 20.7)	0.01
Carbohydrates, total g #	212.9 ± 15.8	212.7 (188.4 - 240.6)	212.2 ± 18.5	211.0 (182.6 - 242.0)	0.598	210.0 ± 17.4	211.8 (179.8 - 237.0)	210.5 ± 15.4	210.0 (185.7 - 236.6)	0.706
Carbohydrates, total %	47.0 ± 3.5	46.9 (41.5 - 53.0)	46.8 ± 4.1	46.6 (40.4 - 53.4)	0.617	46.4 ± 3.8	46.8 (39.7 - 52.4)	46.5 ± 3.4	46.4 (41.1 - 52.3)	0.638
Total sugars g	101.4 ± 16.8	100.6 (71.7 - 129.6)	99.2 ± 16.0	98.9 (72.2 - 125.4)	0.065	93.0 ± 16.5	92.9 (66.5 - 119.5)	94.4 ± 14.6	94.0 (68.9 - 117.9)	0.228
Fat, total g #	21.7 ± 3.9	21.8 (15.0 - 28.2)	21.4 ± 4.0	21.5 (14.9 - 28.0)	0.243	20.8 ± 4.0	20.7 (14.2 - 26.8)	21.4 ± 3.6	21.5 (15.4 - 27.3)	0.019
Fat, total %	69.3 ± 6.4	69.7 (57.5 - 79.0)	69.0 ± 6.5	69.0 (58.2 - 79.9)	0.716	70.3 ± 6.5	70.2 (60.2 - 80.2)	69.1 ± 6.5	69.5 (57.7 - 78.8)	0.099
SFA g #	34.7 ± 3.2	34.8 (28.6 - 39.5)	34.5 ± 3.2	34.5 (29.2 - 39.8)	0.665	35.1 ± 3.3	35.1 (30.1 - 40.2)	34.5 ± 3.2	34.7 (28.8 - 39.4)	0.079
MUFA g #	24.5 ± 4.0	25.0 (17.1 - 30.7)	24.7 ± 4.4	24.5 (17.8 - 32.1)	0.258	25.2 ± 4.0	25.4 (17.9 - 31.3)	25.1 ± 4.8	24.9 (18.1 - 33.4)	0.783
PUFA g #	25.6 ± 4.0	25.7 (18.5 - 31.9)	25.6 ± 4.2	25.5 (18.2 - 32.1)	0.771	27.3 ± 3.9	27.5 (20.9 - 33.2)	27.0 ± 4.2	27.6 (20.2 - 32.8)	0.316
Fiber g #	10.4 ± 1.5	10.2 (8.1 - 13.1)	10.2 ± 1.5	10.1 (7.8 - 12.8)	0.109	10.3 ± 1.7	10.1 (7.9 - 13.2)	10.2 ± 1.6	10.0 (7.9 - 12.9)	0.654
Vitamin A (μg/day) #	15.5 ± 3.2	15.3 (11.3 - 20.0)	15.7 ± 2.9	15.5 (11.7 - 20.8)	0.659	16.4 ± 4.0	16.0 (10.9 - 22.1)	16.8 ± 3.7	16.5 (11.4 - 23.3)	0.282
Vitamin D (μg/day) #	852.6 ± 261.5	802.2 (495.6 - 1.273.6)	927.4 ± 258.9	901.8 (555.1 - 1.313.6)	<0.001	873.9 ± 309.4	831.2 (494.6 - 1.456.4)	895.3 ± 266.9	850.6 (518.2 - 1.375.9)	0.093
Vitamin E (mg TE a/day) #	2.47 ± 1.12	2.30 (1.14 - 4.44)	2.82 ± 1.07	2.70 (1.35 - 4.87)	<0.001	2.42 ± 1.30	2.07 (0.94 - 4.76)	2.56 ± 1.17	2.37 (0.97 - 4.58)	0.02
Thiamin (mg/day) #	8.8 ± 2.4	8.5 (5.6 - 13.2)	9.0 ± 2.3	8.9 (5.8 - 13.2)	0.444	8.9 ± 2.3	8.5 (6.0 - 12.7)	9.0 ± 2.0	8.7 (6.0 - 12.7)	0.755
Riboflavin (mg/day) #	1.20 ± 0.17	1.18 (0.93 - 1.50)	1.27 ± 0.24	1.23 (0.96 - 1.69)	0.001	1.18 ± 0.19	1.15 (0.91 - 1.56)	1.23 ± 0.20	1.19 (0.94 - 1.59)	0.011
Niacin (mg Eq. Niacin/day) #	1.73 ± 0.27	1.74 (1.26 - 2.21)	1.84 ± 0.34	1.79 (1.36 - 2.46)	<0.001	1.67 ± 0.31	1.64 (1.21 - 2.20)	1.77 ± 0.32	1.75 (1.32 - 2.32)	<0.001
Vitamin B6 (mg/day) #	28.9 ± 3.9	28.4 (22.9 - 35.8)	29.5 ± 4.1	29.2 (23.5 - 36.9)	0.069	28.5 ± 4.0	28.2 (22.4 - 36.1)	29.4 ± 4.2	28.9 (23.4 - 37.8)	0.023
Vitamin B12 (μg/day) #	1.81 ± 0.28	1.78 (1.35 - 2.30)	1.89 ± 0.32	1.86 (1.40 - 2.52)	0.022	1.76 ± 0.36	1.69 (1.26 - 2.45)	1.88 ± 0.37	1.87 (1.35 - 2.58)	<0.001
Folate (μg DFE b/day) #	4.2 ± 1.0	4.1 (3.0 - 5.9)	4.2 ± 0.8	4.1 (3.1 - 5.7)	0.401	4.5 ± 1.4	4.3 (2.8 - 6.5)	4.5 ± 1.1	4.4 (2.8 - 6.3)	0.376
Vitamin C (mg/day) #	225.2 ± 44.3	219.8 (161.7 - 305.8)	237.1 ± 47.5	230.1 (175.4 - 321.5)	0.015	229.9 ± 48.5	223.7 (160.4 - 317.6)	243.2 ± 50.4	240.0 (169.3 - 330.1)	0.003
Calcium (mg/day) #	99.5 ± 39.3	92.8 (46.9 - 167.9)	103.7 ± 36.2	98.3 (55.3 - 169.4)	0.249	94.8 ± 37.5	89.9 (44.7 - 167.4)	108.4 ± 40.3	101.9 (54.8 - 182.4)	<0.001
Iron (mg/day) #	949 ± 165	950 (683 - 1.210)	994 ± 168	982 (762 - 1.286)	0.001	923 ± 147	931 (667 - 1.164)	956 ± 152	944 (711 - 1.205)	0.002
Potassium (mg/day) #	11.2 ± 1.8	11.0 (9.0 - 14.7)	11.6 ± 2.1	11.3 (8.9 - 15.9)	0.145	11.3 ± 2.0	11.0 (8.9 - 15.1)	11.5 ± 2.1	11.3 (8.8 - 15.9)	0.682
Sodium (mg/day) #	2.580 ± 282	2.599 (2.081 - 3.073)	2.648 ± 334	2.619 (2.138 - 3.280)	0.003	2.489 ± 345	2.456 (1.960 - 3.072)	2.611 ± 335	2.590 (2.059 - 3.166)	<0.001
Zinc (mg/day) #	1.612 ± 321	1.564 (1.171 - 2.155)	1.636 ± 274	1.620 (1.249 - 2.125)	0.092	1.676 ± 265	1.677 (1.265 - 2.100)	1.698 ± 260	1.680 (1.329 - 2.115)	0.624
Magnesium (mg/day) #	8.6 ± 1.0	8.5 (7.3 - 10.5)	8.7 ± 1.0	8.6 (7.3 - 10.4)	0.316	8.5 ± 1.2	8.3 (6.9 - 10.5)	8.5 ± 1.1	8.4 (7.0 - 10.9)	0.981
Selenium (μg/day) #	237.2 ± 25.9	233.9 (195.5 - 282.6)	241.2 ± 29.7	238.0 (196.0 - 293.9)	0.096	237.6 ± 25.9	236.6 (196.9 - 282.1)	245.2 ± 24.9	245.0 (205.8 - 285.3)	<0.001

	Boys					Girls				
	WORSE QUALITY BREAKFAST		GOOD–QUALITY BREAKFAST			WORSE QUALITY BREAKFAST		GOOD–QUALITY BREAKFAST		
Iodine (μg/day) #	84.0 ± 14.6	84.3 (61.1 - 107.2)	85.1 ± 14.8	84.6 (62.6 - 111.1)	0.368	88.1 ± 13.8	87.9 (67.6 - 109.6)	90.4 ± 16.6	90.0 (66.1 - 117.5)	0.148
Nutrient Rich subscore #	91.5 ± 15.2	89.7 (68.8 - 121.0)	92.0 ± 15.2	89.9 (68.8 - 118.9)	0.39	86.9 ± 14.3	85.8 (66.8 - 112.2)	89.8 ± 15.4	87.6 (67.6 - 117.1)	0.01
LIMiting subscore #	781.1 ± 42.2	795.6 (695.2 - 824.1)	785.2 ± 45.3	804.1 (679.2 - 830.6)	0.001	775.2 ± 51.8	796.8 (670.0 - 827.5)	793.5 ± 36.9	808.6 (706.2 - 827.9)	<0.001
NRF 9.3 score #	284.7 ± 16.6	288.9 (254.2 - 300.0)	283.8 ± 17.4	287.6 (249.3 - 300.0)	0.81	283.6 ± 17.9	286.8 (247.1 - 300.0)	283.1 ± 18.4	286.9 (249.4 - 300.0)	0.941

Nutrients were adjusted for using the residual method for total energy intake except for the percentage of energy from carbohydrates, proteins and fats. The differences have been established by applying t of Student or Mann Whitney (#) between high and low quality breakfast groups according to sex.%E: Percentage of total energy, Niacin was expressed as equivalents of niacin (preformed niacin + tryptophan/60). For vitamin A from β–carotene, a conversion factor of 1/6 was used, whereas for the other carotenoids, a conversion factor of 1/12 was used. Vitamin E was expressed as alpha–tocopherol equivalents (α–TE), and folate intake was calculated as μg of dietary folate equivalents (DFE) (food folate + 1.7 μg synthetic folic acid content of fortified food). NRF9.3 score was calculated based on daily usual intake adjusted by energy of nine nutrients to encourage (Nutrient Rich subscore) and three nutrients to limit (Limiting subscore). A higher NRF 9.3 score is indicative of higher diet quality.

Table S9. Inadequate intakes of macronutrients and micronutrients in Spanish children n (%) by sex and type of breakfast. Results for plausible reporters (n=1311).

	**Boys**							**Girls**						
	**Worse Quality Breakfast**			**Good Quality Breakfast**				**Worse Quality Breakfast**			**Good Quality Breakfast**			
	**< EAR†, n (%)**<br>**[% > UL]**	**< AMDR**	**> AMDR**	**< EAR†, n (%)**<br>**[% > UL]**	**< AMDR**	**> AMDR**	**p value**	**< EAR†, n (%)**<br>**[% > UL]**	**< AMDR**	**> AMDR**	**< EAR†, n (%)**<br>**[% > UL]**	**< AMDR**	**> AMDR**	**p value**
Carbohydrates, total (g/day)	0 (0)			0 (0)			–	0 (0)			0 (0)			–
Protein (g/day)	0 (0)			0 (0)			–	0 (0)			0 (0)			–
Carbohydrates, total (%E)		368 (100)	0 (0)		363 (100)	0 (0)	–		402 (99.7)	0 (0)		332 (100)	0 (0)	0.364
Protein (%E)		0 (0)	2 (0.7)		0 (0)	5 (1.3)	0.249		0 (0)	4 (1)		0 (0)	11 (3.5)	0.026
Fat, total (%E)		0 (0)	157 (42.6)		0 (0)	116 (32)	0.003		0 (0)	189 (46.9)		0 (0)	117 (35.1)	0.001
Vitamin A (µg/day)	5 (1.3)			6 (1.6)			0.744	4 (1)			5 (1.6)			0.526
Vitamin D (µg/day)	368 (100)<br>[0.0]			363 (100)<br>[0.0]			–<br>[-]	402 (99.7)<br>[0.0]			332 (100)<br>[0.0]			0.364<br>[-]
Vitamin E (mg α–TE/day)	102 (27.7)<br>[0.0]			91 (25.2)<br>[0.0]			0.429<br>[-]	156 (38.8)<br>[0.0]			95.5 (28.8)<br>[0.0]			0.004<br>[-]
Thiamin (mg/day)	0 (0)			0 (0)			-	3 (0.6)			0 (0)			0.115
Riboflavin (mg/day)	0 (0)			0 (0)			-	1.3 (0.3)			0 (0)			0.364
Niacin (mg Eq. Niacin/day)	0 (0)			0 (0)			-	0 (0)			0 (0)			-
Vitamin B6 (mg/day)	0 (0)<br>[0.0]			0 (0)<br>[0.0]			-<br>[-]	0 (0)<br>[0.0]			0 (0)<br>[0.0]			-<br>[-]
Vitamin B12 (µg/day)	0 (0)			0 (0)			-	1 (0.3)			0 (0)			0.364
Folate (µg DFE/day)	108 (29.5)<br>[0.6]			76 (20.9)<br>[0.7]			0.008<br>0.986	202 (50)<br>[0.0]			163 (49.2)<br>[0.0]			0.782<br>[-]
Vitamin C (mg/day)	6 (1.6)<br>[0.0]			1 (0.3)<br>[0.0]			0.06<br>[-]	5 (1.3)<br>[0.0]			0 (0)<br>[0.0]			0.042<br>[-]
Calcium (mg/day)	131 (35.5)<br>[0.0]			95 (26.1)<br>[0.0]			0.006<br>[-]	216 (53.7)<br>[0.0]			137 (41.4)<br>[0.0]			0.001<br>[-]
Iron (mg/day)	0 (0)<br>[0.0]			0 (0)<br>[0.0]			-<br>[-]	0 (0)<br>[0.0]			0 (0)<br>[0.0]			-<br>[-]
Potassium (mg/day)†	367 (99.7)			355 (98)			0.031	401 (99.5)			331 (99.8)			0.68
Sodium (mg/day)†	32 (8.7)			26 (7.2)			0.443	48 (11.9)			36 (10.9)			0.661
Zinc (mg/day)	0 (0)			0 (0)			-	12 (2.9)			5 (1.6)			0.189

	Boys							Girls						
	Worse Quality Breakfast			Good Quality Breakfast				Worse Quality Breakfast			Good Quality Breakfast			
	< EAR†, n (%) [% > UL]	< AMDR	> AMDR	< EAR†, n (%) [% > UL]	< AMDR	> AMDR	p value	< EAR†, n (%) [% > UL]	< AMDR	> AMDR	< EAR†, n (%) [% > UL]	< AMDR	> AMDR	p value
	[9.1]			[18.9]			<0.001	[5.8]			[12.5]			0.001
Magnesium (mg/day)	24 (6.5)			38 (10.5)			0.054	62 (15.4)			30 (9)			0.01
Selenium (µg/day)	0 (0)			0 (0)			-	0 (0)			0 (0)			-
	[0.2]			[1.5]			0.055	[1.0]			[1.6]			0.526
Iodine (µg/day)	24 (6.5)			29 (8)			0.444	87 (21.6)			57 (17.2)			0.133
	[0.0]			[0.0]			[-]	[0.0]			[0.0]			[-]

†The chi square test has been applied. EAR: Estimated average requirement, AMDR: Acceptable Macronutrient Distribution Range. UL: Upper limit, ‡Adequate intake data are considered since EAR data are not available.
